# A human two-hit platform modeling post-ischemic sterile inflammation and diastolic dysfunction in hiPSC-derived cardiac models

**DOI:** 10.1016/j.mtbio.2026.103634

**Published:** 2026-09-04

**Authors:** So-Eun Jeong, Choongseong Han, Jong-Hoon Kim, Dong-Hun Woo

**Affiliations:** a Department of Commercializing iPSC Technology, NEXEL Co., Ltd., 8th Floor, 55 Magokdong-ro, Gangseo-gu, Seoul, 07802, South Korea; b Laboratory of Stem Cells and Tissue Regeneration, Department of Biotechnology, College of Life Sciences and Biotechnology, Science Campus, Korea University, 145 Anam-ro, Seongbuk-gu, Seoul, 02841, South Korea

## Abstract

Myocardial ischemia/reperfusion (MI/R) injury exposes the heart to a sequential surge of oxidative stress and sterile inflammation, driving maladaptive remodeling and lethal arrhythmias. However, the human-specific mechanisms linking post-ischemic inflammation to electromechanical dysfunction remain poorly defined. Here, we established a human Two-Hit model using human umbilical vein endothelial cells (HUVECs), human induced pluripotent stem cell-derived cardiomyocytes (hiPSC-CMs), and three-dimensional heart organoids (hHOs). This system integrates acute oxidative priming with the secretome generated by macrophages undergoing secondary necrosis (SN-Sec), thereby recapitulating a redox-active sterile inflammatory microenvironment relevant to the post-ischemic heart. The Two-Hit stress elicited striking cell-type-specific responses. HUVECs exhibited NF-κB activation, exhaustion of inducible antioxidant defenses, and irreversible cell death. In contrast, hiPSC-CMs survived oxidative and inflammatory stress but underwent maladaptive remodeling characterized by stress-induced cytoskeletal remodeling and disruption of gap junction integrity. Mechanistically, sterile inflammation was accompanied by a critical imbalance in calcium handling through selective accumulation of phospholamban (PLN) without a concomitant reduction in SERCA2a expression. The consequent stoichiometric shift markedly correlated with calcium decay kinetics and induced diastolic dysfunction. In hHOs, high-speed optical mapping revealed profound electromechanical discordance, where preserved electrical automaticity became uncoupled from delayed calcium cycling, creating a highly arrhythmogenic substrate. Collectively, these findings identify the ROS-sterile inflammation-PLN axis as a prominent molecular feature correlated with post-ischemic diastolic failure and arrhythmogenesis in human cardiac models. This Two-Hit platform provides a robust framework for dissecting inflammation-driven cardiac remodeling and for evaluating therapeutic strategies targeting post-ischemic electromechanical dysfunction.

## Highlights

• Engineered a human Two-Hit platform modeling post-ischemic sterile inflammation.
• Redox-driven efferocytosis failure triggers toxic secondary necrosis secretomes.
• Secondary necrosis induces electromechanical discordance in human cardiac models.
• Organoids recapitulate adverse ECM deposition and Cx43 remodeling despite vascular preservation.
• A robust screening tool for cardiac therapeutics and redox-toxicity evaluation.

## Graphical abstract

**Figure:**
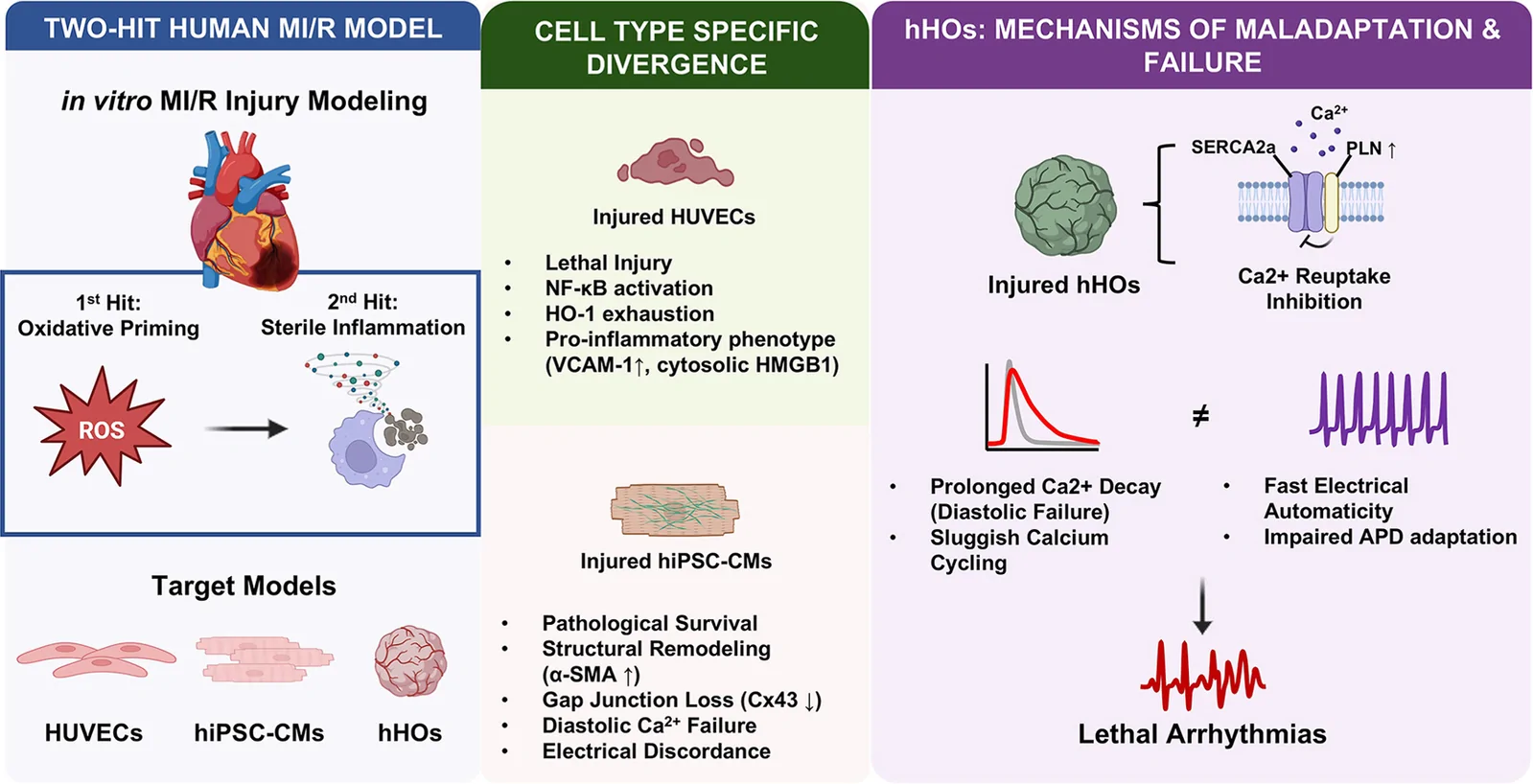

## 1 Introduction

Reperfusion therapies, including percutaneous coronary intervention, have substantially reduced acute mortality following myocardial infarction (MI) [[1], [2], [3]]. Despite these advances, ischemia/reperfusion (I/R) injury remains a key pathological driver of adverse long-term outcomes, particularly heart failure and lethal arrhythmia. This paradox arises because the restoration of blood flow triggers an abrupt burst of reactive oxygen species (ROS) and a sustained sterile inflammatory response, which together promote maladaptive remodeling of the surviving myocardium [[4], [5], [6]]. Given that ischemic necrosis is irreversible, clinical prognosis is critically shaped by the fate of cardiomyocytes and vascular cells that survive the initial ischemic insult during reperfusion [[7], [8], [9], [10]].

Following reperfusion, extensive cell death overwhelms the capacity of local phagocytic systems to efficiently clear apoptotic debris [11,12]. Under these conditions, apoptotic cells undergo secondary necrosis, leading to membrane rupture and the release of damage-associated molecular patterns (DAMPs) [13,14]. This process fundamentally alters the inflammatory landscape by preventing resolution and sustaining a pro-inflammatory M1 macrophage phenotype [15]. Rather than a transient reparative response, failed efferocytosis locks the post-ischemic myocardium into a hostile microenvironment shaped by a pathological inflammatory secretome that prevents resolution and maintains oxidative stress and sterile inflammation [16,17]. Although this inflammatory state is increasingly recognized as a driver of chronic cardiac dysfunction, the mechanisms by which it alters human cardiomyocyte electromechanical integrity remain poorly understood.

Current in vitro models of I/R injury typically rely on single stressors, such as hypoxia mimetics or exogenous oxidants, to reproduce oxidative injury. While these approaches capture aspects of redox imbalance, they fail to recapitulate the immunopathological complexity of the post-ischemic inflammatory milieu [18]. In particular, the contribution of secondary necrosis-driven macrophage secretomes, which reflect frustrated phagocytosis and sustained immune activation, is largely absent from conventional systems. In parallel, animal models present important translational limitations due to species-specific differences in ion channel composition, calcium handling kinetics, and inflammatory signaling pathways [[19], [20], [21]]. These discrepancies complicate the interpretation of arrhythmogenic mechanisms and therapeutic responses in the human heart.

Human induced pluripotent stem cell (hiPSC)-based cardiac platforms offer a powerful alternative for modeling human-specific pathophysiology [[22], [23], [24]]. hiPSC-derived cardiomyocytes enable direct interrogation of calcium cycling and electrophysiological dynamics, while three-dimensional heart organoids integrate multicellular interactions between cardiomyocytes, endothelial cells, and stromal components [25,26]. Importantly, these systems provide an opportunity to examine how inflammatory cues reshape cardiac function across distinct structural contexts, from single-cell monolayers to tissue-level architectures [27].

In the present study, we established a human-relevant Two-Hit model that sequentially combines oxidative priming with sterile inflammation derived from macrophages exposed to secondary necrosis. This approach is designed to mimic key features of the post-ischemic microenvironment that arise when efferocytosis fails. Using HUVECs, hiPSC-derived cardiomyocytes, and human heart organoids, we systematically examined how this redox-inflammatory axis influences cell survival, structural remodeling, calcium handling, and electrophysiological stability. Specifically, we identified a critical mechanism where the inflammatory secretome disrupts calcium homeostasis through a selective upregulation of phospholamban (PLN) without compensatory SERCA2a reduction. This stoichiometric imbalance drives diastolic dysfunction and electromechanical discordance, highlighting the ROS-inflammation-PLN axis as a potential candidate for therapeutic intervention against post-ischemic diastolic failure.

## 2 Materials and methods

### 2.1 Antibodies and reagents

tert-Butyl hydroperoxide (t-BHP, 458139), Cobalt(II) chloride (CoCl_2_, 232696), phorbol 12-myristate 13-acetate (PMA, P1585), 2′,7′-Dichlorofluorescin diacetate (DCFDA, D6883), Lipopolysaccharides from *Escherichia coli* O26:B6 (LPS, L5543) and Bovine Serum Albumin (BSA, A9647) were purchased from Sigma-Aldrich. Calcein AM (C34852), Di-4-ANEPPS (D1199), Fluo-4 AM (F14201), Dead Cell Apoptosis Kits with Annexin V for Flow Cytometry (V13245) and MitoSOX™ Red Mitochondrial Superoxide Indicator (M36008) were obtained from Invitrogen. Recombinant Human IFN-gamma Protein (285-IF-100) and Proteome Profiler Human Cytokine Array Kit (ARY005B) were purchased from R&D systems. Cell Counting Kit-8 (CCK-8, CK04-13) was purchased from Dojindo.

Primary antibodies against phospho-p65 (3033S), p65 (8242S), HO-1 (5853S), phospho-p38 (9211S), p38 (9212S), phospho-PLN (8496S), PLN (14562S), SERCA2a (9580S), α-SMA (19245S), and HIF-1α (14179S) were obtained from Cell Signaling Technology. Primary antibodies against GCLC (ab53179), GCLM (ab126704), VCAM-1 (ab134047), HMGB1 (ab18256), cTNT (ab8295), Cx43 (ab11370), and Fibronectin (ab268020) were purchased from Abcam. Primary antibody against β-actin (PA1-183) was obtained from Invitrogen. Primary antibody against CD31 (AF806) was purchased from R&D Systems. Secondary antibodies conjugated with HRP or Alexa Fluor were purchased from Invitrogen.

### 2.2 Culture of human monocytic and T-cell lines

THP-1 and Jurkat cells were cultured in RPMI-1640 containing 10% FBS, 1% penicillin-streptomycin, and 0.05 mM 2-mercaptoethanol (for THP-1 only) under standard conditions. Comprehensive details regarding cell line specifications and maintenance protocols are provided in the Supplementary Methods.

### 2.3 M1 macrophage differentiation and activation

THP-1 Differentiation and M1 Polarization THP-1 monocytes were differentiated into macrophages by incubation with 100 ng/mL PMA for 48 h. Following a wash step to remove residual PMA, M1 polarization was induced by treating the differentiated cells with 20 ng/mL recombinant human IFN-γ and 100 pg/mL LPS for an additional 48 h. Phenotypic transition was verified by observing morphological changes. Full details of the polarization protocol and morphological validation are available in the Supplementary Methods.

### 2.4 Generation of secondary necrosis-conditioned medium

To mimic the sterile inflammatory microenvironment of failed efferocytosis, we developed a secondary necrosis-driven model. Briefly, Jurkat T cells were treated with 100 μM t-BHP for 24 h to induce secondary necrosis. These necrotic cells were collected by centrifugation and subsequently co-cultured with THP-1-derived M1 macrophages for an additional 24 h. Finally, the co-culture suspension was centrifuged, and the supernatant was collected and defined as SN-Sec (Secondary Necrosis-Secretome). Conditioned media harvested separately from apoptotic cells alone or M1 macrophages alone were defined as AC-Sec (Apoptotic Cell Secretome) and M1-Sec (M1 Macrophage Secretome), respectively.

### 2.5 HUVEC culture and maintenance

Human umbilical vein endothelial cells (HUVECs, ATCC, CRL-1730) were maintained in F-12K medium supplemented with 10% FBS, 0.1 mg/mL heparin, and 0.05 mg/mL endothelial cell growth supplement (ECGS). Cells were cultured under standard conditions and subcultured using TrypLE when reaching 80% confluence. Detailed formulations and subculturing procedures are provided in the Supplementary Methods.

### 2.6 Culture and maintenance of human iPSC-derived cardiomyocytes

Commercially available human induced pluripotent stem cell-derived cardiomyocytes (hiPSC-CMs; Cardiosight®-S, NEXEL Co., Ltd., Seoul, Korea) were used for experiments. The cells were thawed and maintained strictly following the manufacturer's protocols (Cardiosight®-S User Guide). Briefly, the hiPSC-CMs were plated onto Matrigel-coated (BD Biosciences) culture vessels at a density of 1 × 10^5^ cells/cm^2^ using the Cardiosight®-S Plating Medium. After 24 h of incubation at 37 °C in 5% CO^2^, the plating medium was replaced with Cardiosight®-S Maintenance Medium. The maintenance medium was subsequently refreshed every 48 h. The cells were allowed to recover and stabilize for at least 7 days post-thawing until they exhibited robust, spontaneous, and synchronous rhythmic contractions before being subjected to experimental Two-Hit stress.

### 2.7 Generation and differentiation of human heart organoids

Human iPSC derived heart organoids (hHOs) were generated according to our previous report [25]. Briefly, hHOs differentiation was initiated approximately 4 days after seeding of hiPSCs and performed through small-molecule control of the Wnt/β-catenin pathway with 6 μM GSK3 inhibitor in RPMI 1640 supplemented with B-27 without insulin for mesoderm induction. The differentiated mesoderm was subsequently cultured with 30 ng/mL of BMP, 30 ng/mL of VEGF, 30 ng/mL of FGF, and 10 μM TGF-beta/Smad inhibitor-containing medium from D8 to D14 to induce differentiation into cardiomyocytes, endothelial cells, and fibroblasts. Following this induction phase, the hHOs were transitioned to a maturation medium consisting of RPMI 1640 supplemented with B-27 without vitamin A, 30 ng/mL VEGF, 30 ng/mL FGF2, and 10 mM SB431542, notably omitting BMP4 to allow for the stable terminal differentiation of the lineages. The medium was refreshed every 48 h. Mature hHOs cultured for 30 days or longer (>D30) were utilized for all subsequent experimental procedures.

### 2.8 The two-hit ischemia/reperfusion model

We established a sequential stress model recapitulating MI/R injury across three distinct platforms: HUVECs, 2D hiPSC-CM monolayers, and 3D hHOs. To mimic acute oxidative stress (Hit 1), HUVECs and hiPSC-CMs were pulsed with t-BHP (100 μM and 1 mM, respectively) for 20 min, whereas 3D hHOs were treated with 100 μM CoCl_2_ for 3 h. Subsequently, the medium was replaced with SN-Sec (Hit 2) to model the reperfusion-associated inflammatory remodeling phase. The exposure duration was optimized for each model: 48 h for HUVECs, 96 h for hiPSC-CMs, and 6 days for hHOs.

### 2.9 Annexin V-FITC/PI staining for apoptosis analysis

Cell viability and apoptosis were quantified using the Annexin V-FITC/PI Apoptosis Detection Kit according to the manufacturer's instructions. Samples were analyzed using an ACEA NovoCyte-3000 flow cytometer (Agilent). Cell populations were categorized into Annexin V+, and PI + populations. Data were processed using NovoCyte software (Agilent). Detailed staining protocols and flow cytometry settings are described in the Supplementary Methods.

### 2.10 Measurement of intracellular and mitochondrial ROS

To evaluate the levels of total intracellular ROS and mitochondrial superoxide, DCFDA and MitoSOX Red were utilized, respectively.

#### 2.10.1 Flow cytometry analysis for 2D cultured cells

For the quantitative assessment of ROS in 2D-cultured cells (e.g., HUVECs or hiPSC-CMs), cells were incubated with 10 μM DCFDA or 5 μM MitoSOX at 37 °C for 30 min in the dark. After staining, cells were detached using TrypLE™ and harvested. Then, the cells were washed twice with cold PBS and immediately analyzed using a flow cytometer.

#### 2.10.2 Fluorescence imaging for human heart organoids

For the 3D heart organoids, the staining process was performed in situ. The hHOs were incubated with 10 μM DCFDA or 5 μM MitoSOX for 30 min at 37 °C. After staining, the organoids were washed three times with fresh Tyrode's buffer to minimize background fluorescence. Live-cell fluorescence imaging was performed while the organoids were submerged in Tyrode's buffer using a Nikon Eclipse Ti2 microscope. The fluorescence intensity was quantified using Fiji (ImageJ) software by measuring the integrated density of the organoid areas.

### 2.11 Cytokine profiling using antibody array

Cytokine Profiling Secreted cytokines in culture supernatants were analyzed using the Proteome Profiler Human Cytokine Array Kit according to the manufacturer's instructions. Protein signals were captured using a ChemiDoc imaging system (Bio-Rad) and quantified by pixel intensity using ImageJ software. All data were normalized to the internal reference spots after background subtraction. Detailed procedures for membrane incubation and signal processing are provided in the Supplementary Methods.

### 2.12 Western blot analysis

Total protein was extracted using RIPA buffer and quantified via Bradford assay. Equal amounts of protein were separated by SDS-PAGE and transferred to nitrocellulose membranes. After blocking with 3% BSA, membranes were incubated with primary antibodies overnight at 4 °C, followed by HRP-conjugated secondary antibodies. Bands were visualized using an ECL system and quantified via ImageJ, with expression levels normalized to loading controls. Detailed antibody information and full-length blots are available in the Supplementary Methods.

### 2.13 Cell viability assay

Cell viability was determined using the CCK-8 assay kit according to the manufacturer's instructions. Absorbance was measured at 450 nm using a SpectraMax iD3 microplate reader (Molecular Devices). Results were expressed as a percentage of the untreated control group. Detailed incubation times and calculation procedures are described in the Supplementary Methods.

### 2.14 Preparation and histological sectioning of heart organoids

hHOs were fixed in 4% PFA and cryoprotected in 30% sucrose. The organoids were then embedded in OCT compound and sectioned at a thickness of 10 μm using a cryostat (Leica CM1860). Detailed protocols for fixation, cryoprotection, and sectioning are available in the Supplementary Methods.

### 2.15 Immunofluorescence staining and fluorescence imaging

Samples were fixed with 4% PFA and incubated with primary antibodies overnight at 4 °C after blocking and permeabilization with 1% BSA and 0.5% Triton X-100. Signals were detected using Alexa Fluor-conjugated secondary antibodies, with DAPI for nuclear counterstaining. Fluorescence images were captured using a Nikon Eclipse Ti2 microscope (Nikon), whereas high-resolution confocal images and Z-stacks were acquired using an LSM 900 confocal microscope (Carl Zeiss). Images were quantified using Fiji (ImageJ) software. Detailed staining protocols and antibody specifications are provided in the Supplementary Methods.

### 2.16 Functional optical mapping of calcium and voltage dynamics

Electrophysiological properties of hiPSC-CMs and hHOs were evaluated using the Nautilai system (CuriBio). Calcium and membrane potential dynamics were measured using 10 μM Fluo-4 AM and 5 μM Di-4-ANEPPS (Invitrogen), respectively, in Tyrode's buffer. High-speed fluorescence images were captured and analyzed using Nautilai software (CuriBio). Comprehensive staining protocols and imaging parameters are detailed in the Supplementary Methods.

### 2.17 Live/dead cell viability assay and imaging

The viability of hHOs was visualized using 1 μM of Calcein-AM and PI. Fluorescence images were captured using a Nikon Eclipse Ti2 microscope (Nikon) with organoids submerged in Tyrode's buffer to maintain structural integrity. Viability was quantified by calculating the ratio of Calcein-positive (live) to PI-positive (dead) areas using Fiji (ImageJ) software. Detailed incubation and imaging procedures are provided in the Supplementary Methods.

### 2.18 Statistical analysis

All statistical analyses were performed using GraphPad Prism software (Version 10.6.1, GraphPad Software). Data are presented as mean ± standard error of the mean (SEM) of at least three independent biological replicates. For comparisons between two groups, an unpaired two-tailed Student's t-test was used. For comparisons among three or more groups (e.g., Control, One-Hit, and Two-Hit), statistical significance was determined using one-way analysis of variance (ANOVA) followed by Tukey's multiple comparison test. A P-value of < 0.05 was considered statistically significant. For the Ca2+ transient irregularity analysis (Fig. 6A), outliers were identified and excluded using the ROUT method (Q = 1%) implemented in GraphPad Prism.

## 3 Results

### 3.1 Establishment of a secondary necrosis–derived inflammatory secretome that mimics post-ischemic sterile inflammation

**Fig. 1:**
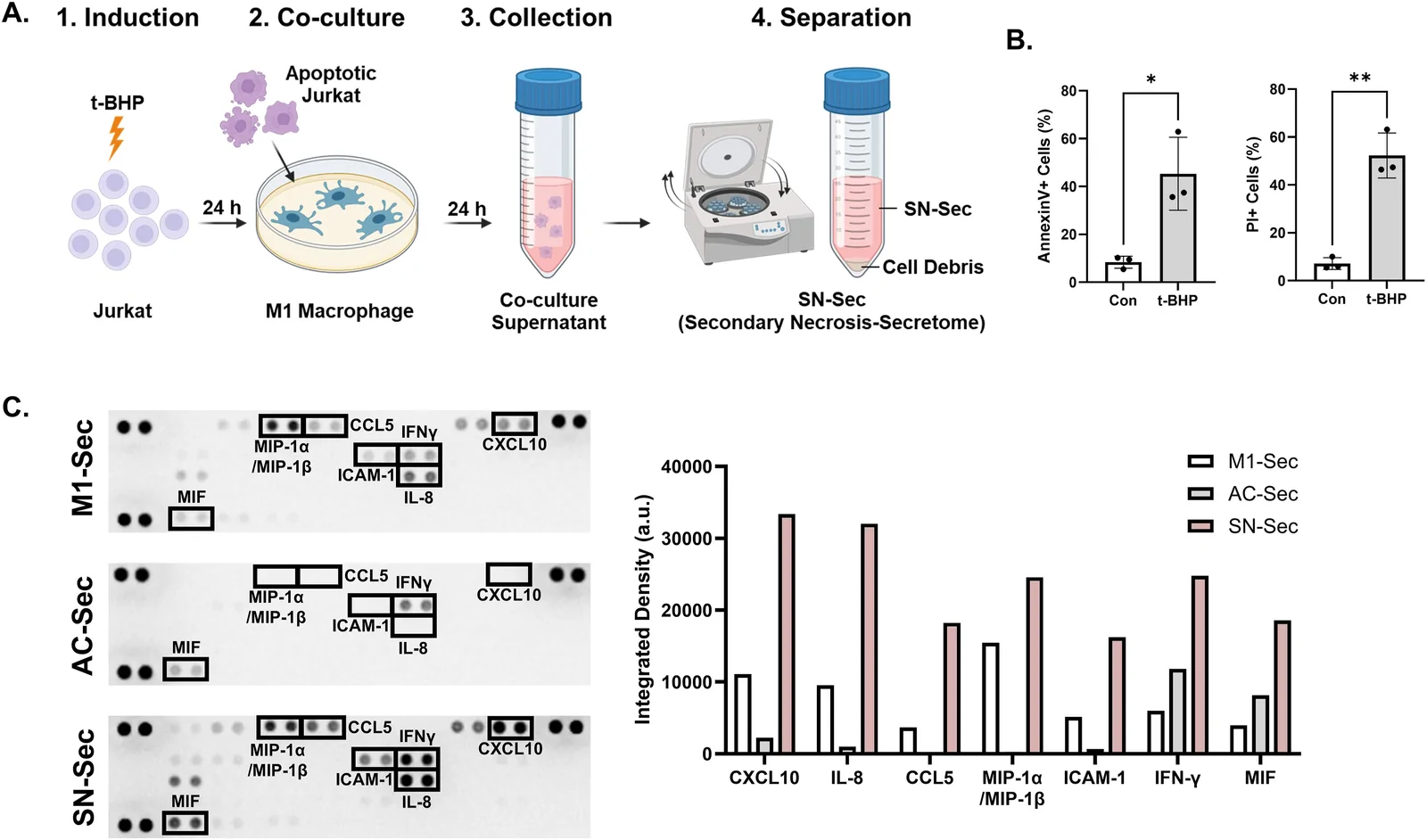
**Establishment and characterization of SN-Sec as an in vitro mimic of sterile inflammation in MI/R injury.** **A.** Schematic illustration of the experimental strategy. To replicate the inflammatory microenvironment of MI/R injury, M1 macrophages were co-cultured with t-BHP-induced apoptotic Jurkat cells. The resulting cell-free supernatant, termed secondary necrosis-secretome (SN-Sec), was collected to be used as a sterile inflammation mimic. Conditioned media from apoptotic cells alone (AC-Sec) and M1 macrophages alone (M1-Sec) were collected as controls. **B.** Quantitative assessment of membrane permeabilization. Flow cytometric analysis of Jurkat cells treated with t-BHP using Annexin V/PI staining. The percentages of early apoptotic (Annexin V+) and secondary necrotic/necrotic (PI+) populations were quantified separately to verify loss of membrane integrity, demonstrating that the treated population robustly enters secondary necrosis during the generation of SN-Sec. Data are presented as Mean ± SEM from 3 independent experiments. **P < 0.01 vs. Control. **C.** Cytokine array profiling. The bar graph represents the densitometric quantification of the array spots.

To model the sterile inflammatory microenvironment arising from failed efferocytosis during myocardial ischemia/reperfusion (MI/R) injury, we generated secondary necrosis-secretome (SN-Sec) using a macrophage-necrotic cell co-culture system (Fig. 1A). To establish the SN-Sec stimulus, we first initialized the cell death cascade in Jurkat T cells using 100 μM t-BHP. Flow cytometric analysis using Annexin V and Propidium Iodide (PI) staining confirmed not only a significant induction of early apoptotic cells (Annexin V+) but also a massive increase in total PI + secondary necrotic cells (Fig. 1B). This pronounced accumulation of PI + cells directly demonstrates the loss of cellular membrane integrity, confirming that the challenged cells successfully progressed to secondary necrosis. These apoptotic cells were subsequently co-cultured with M1-polarized macrophages derived from THP-1 monocytes, whose polarization was validated by increased expression of canonical M1 markers (Supplementary Fig. 1). After co-culture, the cell-free supernatant was collected and defined as SN-Sec. Conditioned media harvested separately from apoptotic cells alone (AC-Sec) or M1 macrophages alone (M1-Sec) served as controls.

Cytokine profiling revealed that SN-Sec exhibited a markedly amplified inflammatory signature compared with either AC-Sec or M1-Sec (Fig. 1C). While individual control media showed only basal or modest cytokine levels, SN-Sec displayed a synergistic induction of multiple inflammatory mediators that act as redox-sensitive drivers. Quantitative analysis identified robust up-regulation of CXCL10, IL-8, CCL5, and MIP-1α/β, indicating strong leukocyte recruitment potential. In parallel, ICAM-1 levels were markedly elevated, consistent with an activated inflammatory milieu capable of promoting endothelial-immune cell interactions. IFN-γ and macrophage migration inhibitory factor (MIF) were also significantly increased in SN-Sec. Notably, despite equivalent IFN-γ supplementation during macrophage polarization, IFN-γ levels were selectively amplified in SN-Sec, suggesting enhanced inflammatory signaling induced by secondary necrosis.

A broader analysis of the secretome further supported this synergistic inflammatory profile. Heatmap visualization demonstrated a global increase in inflammatory mediators uniquely associated with SN-Sec (Supplementary Fig. 2A; Supplementary Table 1). Individual factor analysis confirmed additional elevation of chemokines and regulatory cytokines, including CCL2, CXCL1, CXCL11, and IL-1 receptor antagonist (IL-1ra) (Supplementary Fig. 2B). The concurrent presence of pro-inflammatory and compensatory factors indicates that SN-Sec recapitulates a complex inflammatory environment rather than a unidirectional cytokine response. Collectively, these data establish SN-Sec as a physiologically relevant in vitro surrogate of the sterile inflammatory milieu generated by efferocytotic failure.

### 3.2 Two-hit stress induces endothelial antioxidant exhaustion and inflammatory activation

**Fig. 2:**
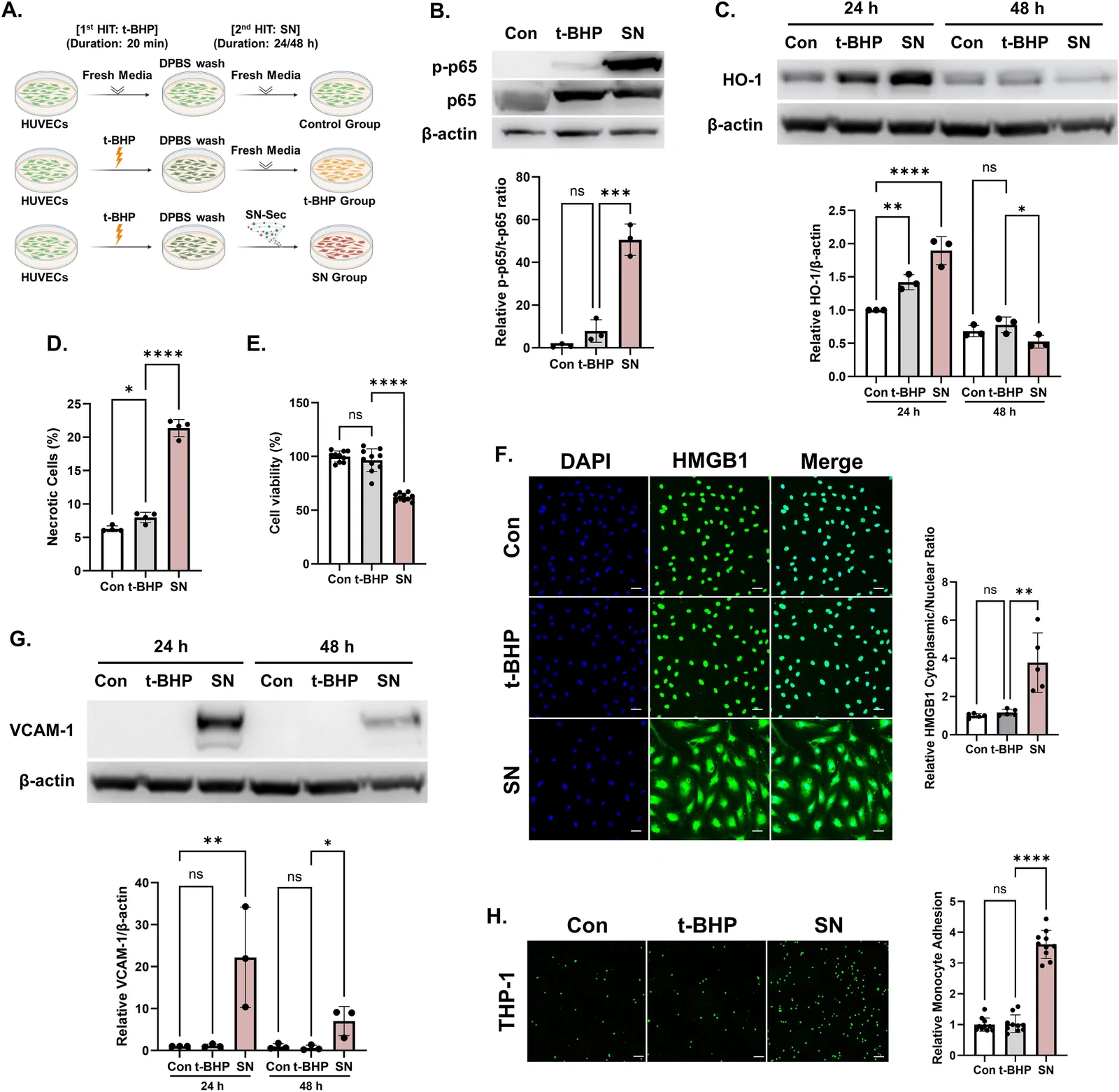
**Characterization of endothelial viability, inflammatory signaling, and leukocyte recruitment under Two-Hit stress conditions.** **A.** Schematic illustration of experimental design. HUVECs were treated with fresh media (Control), t-BHP followed by fresh media (One-Hit; t-BHP), or t-BHP followed by SN-Sec (Two-Hit; SN). **B.** Western blot analysis and quantification of phosphorylated p65 (p-p65) relative to total p65 at 15 min post-treatment. **C.** Western blot analysis of HO-1 expression at 24 h and 48 h (normalized to β-actin). **D.** Flow cytometric analysis of cell death using Annexin V/PI staining at 48 h. **E.** Cell viability assessed by CCK-8 assay at 48 h. **F.** Representative immunofluorescence images of HMGB1 (Green) and DAPI (Blue) at 48 h. The bar graph indicates the Cytoplasmic-to-nuclear ratio of HMGB1 fluorescence intensity. **G.** Western blot analysis of VCAM-1 expressions at 24 h and 48 h (normalized to β-actin). **H.** Monocyte attachment assay. Representative fluorescence micrographs showing the adhesion of THP-1 monocytes (labeled with calcein-AM, green) to HUVEC monolayers following 1 h of co-incubation. Images are representative of these experiments. ns: not significant, *P < 0.05, **P < 0.01, ***P < 0.001, ****P < 0.0001 vs. corresponding t-BHP. Scale bar: 100 μm.

Because vascular endothelial cells are among the earliest targets exposed to reperfusion-associated oxidative and inflammatory stress [[28], [29], [30]], we first examined the effects of the Two-Hit paradigm in HUVECs (Fig. 2A). Cells were primed with t-BHP to induce acute oxidative stress and subsequently exposed to SN-Sec to model sterile inflammation. Treatment with t-BHP alone followed by fresh medium served as the One-Hit group. Oxidative priming was confirmed by a significant increase in DCFDA-positive cells following t-BHP exposure (Supplementary Fig. 3A).

We initially assessed activation of NF-κB signaling, a central redox-sensitive inflammatory pathway [31,32]. While t-BHP treatment increased total p65 protein levels, it did not induce significant phosphorylation. In contrast, the SN-Sec treated group exhibited a pronounced increase in phosphorylated p65, indicating robust NF-κB activation under Two-Hit conditions (Fig. 2B). We next evaluated the inducible antioxidant response, focusing on heme oxygenase-1 (HO-1), a pivotal enzyme in redox homeostasis [[33], [34], [35]]. After 24 h, the SN-Sec treated group showed a modest but significant increase in HO-1 expression relative to the t-BHP group. However, this response was not sustained and the HO-1 levels in the SN-Sec treated group declined sharply after 48 h, falling below those observed in the t-BHP group (Fig. 2C). This decline was specific to HO-1, as expression of the constitutive antioxidant enzymes GCLC and GCLM remained unchanged across all groups and time points (Supplementary Fig. 4A), indicating a selective failure of the antioxidant defense rather than a global suppression.

Consistent with impaired redox defense, endothelial viability was markedly compromised under Two-Hit stress. Flow cytometric analysis revealed a substantial increase in apoptotic and necrotic cells in the SN-Sec treated group at 48 h, whereas the t-BHP group showed only a modest increase (Fig. 2D). This was corroborated by CCK-8 assays, which showed a significant reduction in cell viability in the SN-Sec treated group compared with the t-BHP group (Fig. 2E). Immunofluorescence analysis further demonstrated nucleocytoplasmic translocation of HMGB1, a critical nuclear protein that acts as a redox-active DAMP [36,37], in the SN-Sec treated group, whereas it remained predominantly nuclear in the control and t-BHP groups (Fig. 2F). This redistribution suggests that Two-Hit stress induced the loss of nuclear integrity and the release of damage-associated inflammatory signals in endothelial cells.

Finally, we examined endothelial activation relevant to leukocyte recruitment. The expression of VCAM-1, a key adhesion molecule mediating monocyte infiltration [38,39], was negligible in the control and t-BHP groups at both 24 and 48 h. In contrast, the SN-Sec treated group showed robust VCAM-1 expression at 24 h, which persisted at 48 h (Fig. 2G). A lower-molecular-weight VCAM-1 band was also detected in the SN-Sec treated group, suggesting stress-associated proteolytic processing. Functionally, this molecular activation translated into enhanced immune cell interaction, as THP-1 monocyte adhesion to endothelial monolayers in the SN-Sec treated group was significantly increased compared with the t-BHP group.

Together, these results demonstrate that oxidative stress alone is insufficient to induce sustained endothelial dysfunction. Instead, the combination of oxidative priming and a secondary necrosis–derived inflammatory secretome drives antioxidant exhaustion, endothelial collapse, and leukocyte recruitment under Two-Hit conditions.

### 3.3 Two-hit stress allows hiPSC-derived cardiomyocytes to survive but induces maladaptive structural remodeling

**Fig. 3:**
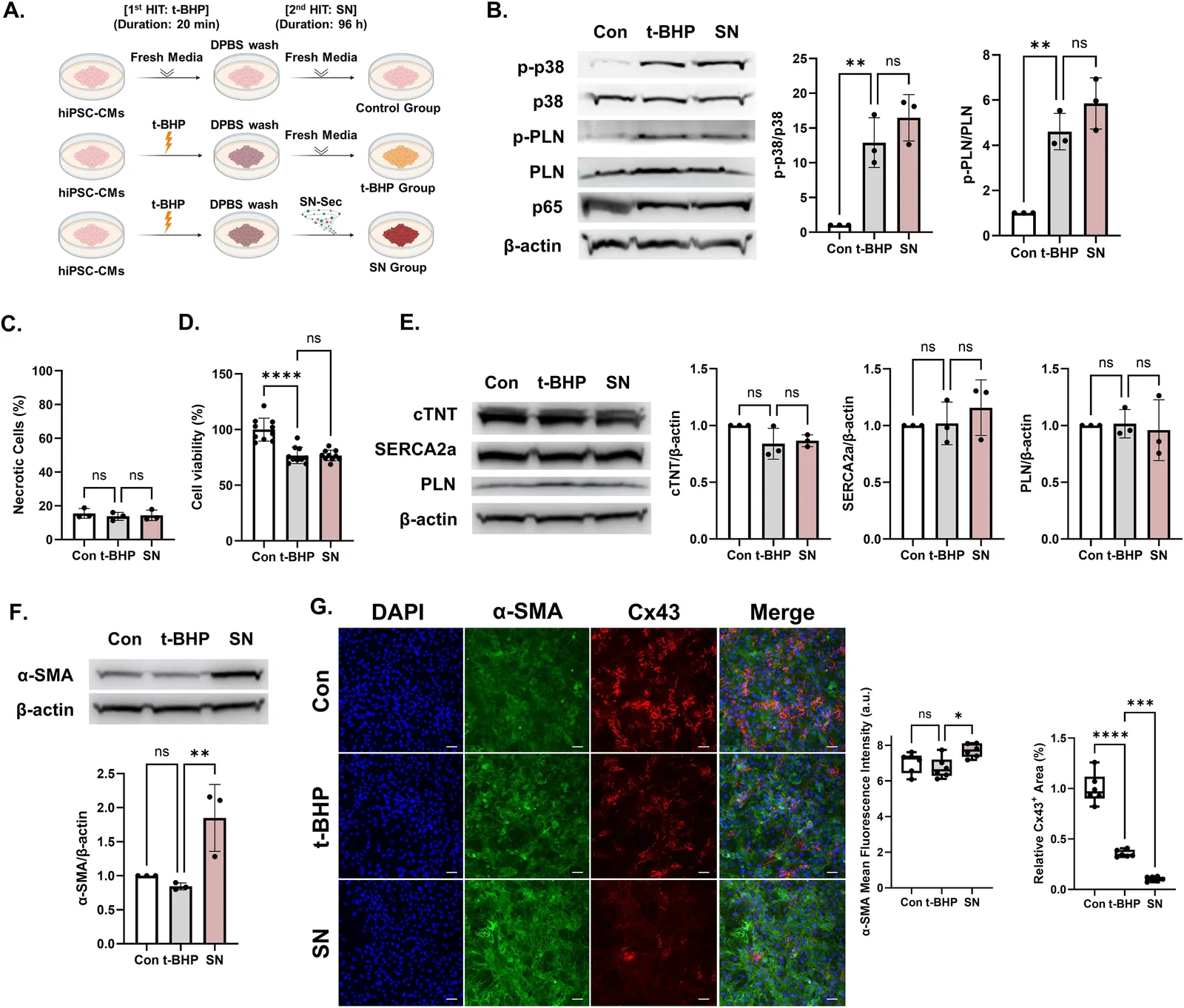
**Analysis of viability, stress-induced cytoskeletal remodeling, and gap junction integrity in human iPSC-derived cardiomyocytes under Two-Hit stress conditions.** **A.** Schematic illustration of experimental design. Human iPSC-derived cardiomyocytes (hiPSC-CMs) were treated with fresh media (Control), t-BHP followed by fresh media (One-Hit; t-BHP), or t-BHP followed by SN-Sec (Two-Hit; SN). **B.** Western blot analysis of stress signaling markers (p-p38, p38, p-PLN, PLN, p65) relative to their total proteins at 30 min post-treatment. **C.** Flow cytometric analysis of cell death using Annexin V/PI staining at 96 h. **D.** Cell viability assessment by CCK-8 assay at 96 h. **E.** Western blot analysis of cardiac structural (cTNT) and calcium handling proteins (SERCA2a, PLN) at 96 h as a marker of stress-induced cytoskeletal remodeling or partial dedifferentiation (normalized to β-actin). **F.** Western blot analysis of α-SMA expression at 96 h (normalized to β-actin). **G.** Representative immunofluorescence images of α-SMA (Green, indicating cytoskeletal remodeling), Cx43 (Red), and DAPI (Blue) at 96 h. Bar graphs show the quantification of α-SMA fluorescence intensity and % Area of Cx43 puncta per field. Data are presented as Mean ± SEM from 3 independent experiments. Images are representative of these experiments. ns: not significant, *P < 0.05, **P < 0.01, ***P < 0.001, ****P < 0.0001 vs. t-BHP. Scale bar: 100 μm.

Given the pronounced vulnerability of endothelial cells to Two-Hit stress, we next examined whether hiPSC-CMs exhibit a distinct response profile. hiPSC-CMs were subjected to oxidative priming followed by exposure to SN-Sec using our Two-Hit system (Fig. 3A). Consistent with their intrinsic antioxidant capacity [40], hiPSC-CMs displayed markedly greater resistance to oxidative stress than HUVECs. Specifically, exposure to 100 μM t-BHP, which robustly induced ROS in endothelial cells (Supplementary Fig. 3), failed to elicit significant mitochondrial superoxide generation in hiPSC-CMs (Supplementary Fig. 5). Escalation of t-BHP concentration to 1 mM was required to induce a measurable increase in MitoSOX-positive cells, confirming successful oxidative priming under the One-Hit conditions (Supplementary Fig. 5).

Under this condition, we first assessed acute stress signaling responses following short-term exposure to t-BHP. In contrast to endothelial cells, hiPSC-CMs did not exhibit canonical NF-κB activation. Instead, oxidative priming induced robust activation of the stress-responsive kinase p38 MAPK [41], as evidenced by an increased p-p38 (phosphorylated p38)/p38 ratio (Fig. 3B). This response was observed under both One-Hit and Two-Hit conditions. In parallel, phosphorylation of PLN, a regulator of Ca^2+^-ATPase (SERCA2a) [42], at Ser16/Thr17 was significantly increased in both groups, indicating an early adaptive response for modulating calcium handling during oxidative stress (Fig. 3B).

Despite sustained exposure to the inflammatory secretome, hiPSC-CMs maintained viability over prolonged culture. Flow cytometric analysis at 96 h of treatments revealed no significant increase in necrotic cell populations in either the t-BHP or SN-Sec treated group (Fig. 3C). Although overall cell viability was modestly reduced by t-BHP or SN-Sec, as measured by CCK-8 assay, cell survival rates between the two groups remained largely preserved (Fig. 3D). Consistent with this, the expression levels of key structural and calcium-handling proteins, including cardiac troponin T (cTNT), SERCA2a, and total PLN, were maintained across all experimental groups (Fig. 3E).

While the survival of hiPSC-CMs was preserved, Two-Hit stress induced distinct features of pathological remodeling. Analysis of α-smooth muscle actin (α-SMA), a marker often reactivated during embryonic heart development and structural remodeling, revealed a selective increase in expression in the SN-Sec treated group, whereas the t-BHP group did not alter α-SMA levels (Fig. 3F). Immunofluorescence staining confirmed a significant enhancement of α-SMA signal intensity specifically under Two-Hit conditions (Fig. 3G), indicating pronounced cytoskeletal remodeling or a regression toward a partial dedifferentiated state under Two-Hit conditions. We further examined the integrity of gap junctional coupling by assessing Connexin 43 (Cx43) expression. The t-BHP group showed a measurable reduction in Cx43 levels relative to the control group. Importantly, exposure to SN-Sec further exacerbated this loss, resulting in a pronounced depletion of Cx43 protein under Two-Hit stress (Fig. 3G). This disruption of gap junction integrity occurred despite preserved cell survival, highlighting a dissociation between viability and electromechanical connectivity in hiPSC-CMs.

Collectively, these data demonstrate that hiPSC-CMs remain viable under oxidative and inflammatory stress without undergoing overt cell death. However, survival under Two-Hit conditions is accompanied by maladaptive structural remodeling, characterized by fibrotic marker induction and severe impairment of gap junction integrity. This phenotype reflects a transition toward a cardiomyocyte state that preserves viability while exhibiting functional impairment under sterile inflammatory stress.

### 3.4 Two-hit stress disrupts calcium handling and electrophysiological stability in hiPSC-derived cardiomyocytes

**Fig. 4:**
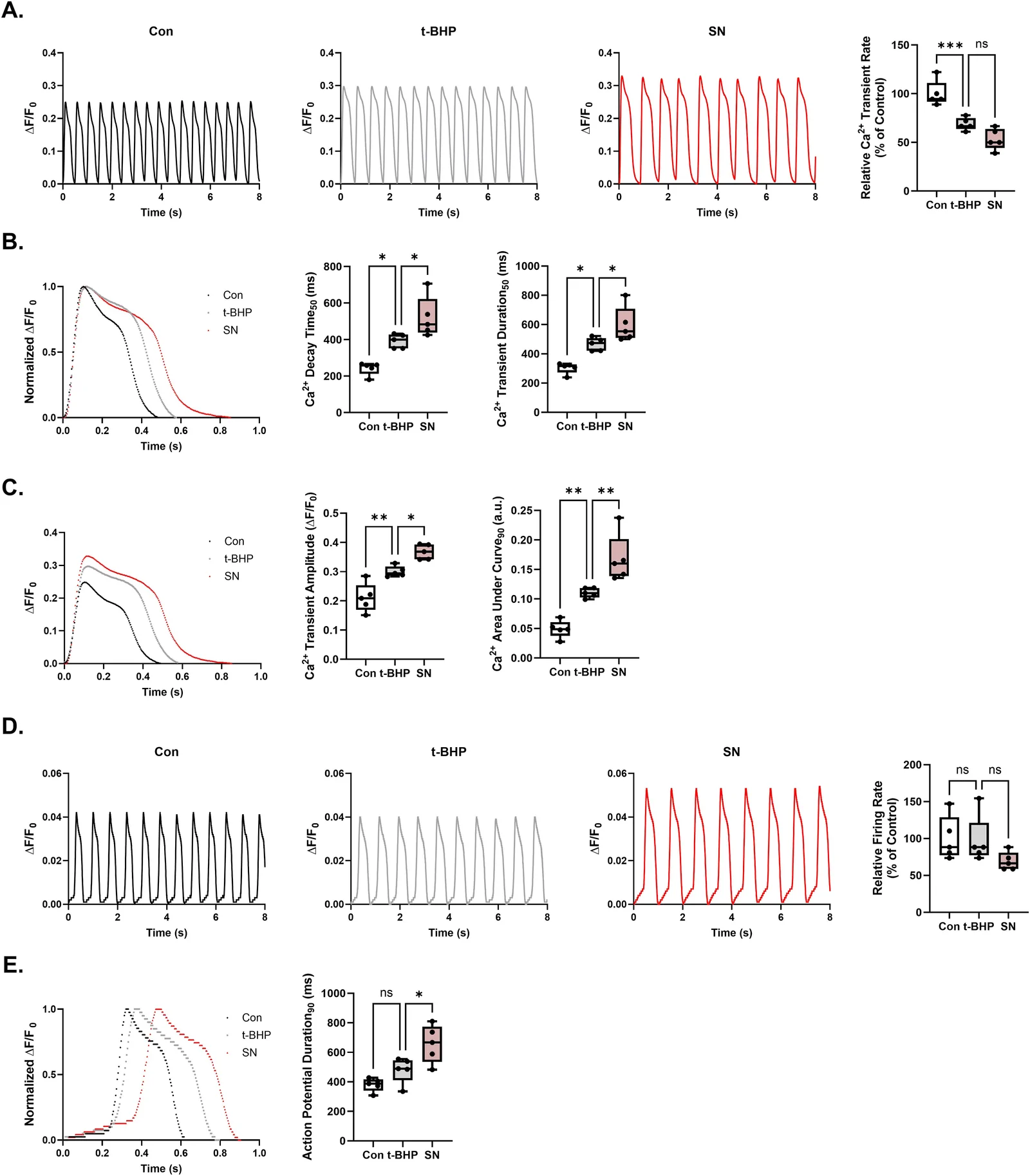
**Assessment of calcium handling dynamics and electrophysiological properties in human iPSC-derived cardiomyocytes under Two-Hit stress conditions.** **A.** Representative spontaneous calcium transient traces (ΔF/F_0_) recorded in human iPSC-derived cardiomyocytes (hiPSC-CMs) using Fluo-4 AM over an 8 s duration. The bar graph displays the relative Ca^2+^ transient rate in beats per minute (BPM). **B.** Representative normalized single transient trace. Bar graphs quantify the Ca^2+^ decay time_50_ and Ca^2+^ transient duration_50_ (CTD_50_). **C.** Representative single raw transient trace (ΔF/F_0_). Bar graphs display the Ca^2+^ transient amplitude (ΔF/F_0_) and the Ca^2+^ area under the curve_90_ (AUC_90_). **D.** Representative optical action potential traces (ΔF/F_0_) recorded using Di-4-ANEPPS over an 8 s duration. The bar graph displays the firing rate in beats per minute (BPM). **E.** Representative normalized single action potential trace. The bar graph quantifies the action potential duration at 90% repolarization (APD_90_). Data are presented as Mean ± SEM from 5 independent experiments. Traces are representative of these experiments. ns: not significant, *P < 0.05, **P < 0.01, ***P < 0.001, vs. t-BHP.

Stress-induced cytoskeletal remodeling and loss of Cx43 are known to impair intrinsic contractility and electrical synchrony between cardiomyocytes [[43], [44], [45]]. To determine whether the structural remodeling observed under Two-Hit stress translates into functional impairment, we assessed intracellular calcium dynamics and electrophysiological properties in hiPSC-CMs using high-speed optical mapping (Fig. 4A). Calcium transients were recorded with Fluo-4 AM, and membrane potential dynamics were evaluated using the voltage-sensitive dye Di-4-ANEPPS.

Under control conditions, hiPSC-CMs exhibited regular spontaneous calcium oscillations with stable beating rhythms. Oxidative priming induced a reduction in spontaneous beating rate, and this trend was further accentuated following exposure to SN-Sec, although the difference between the t-BHP and SN-Sec treated groups did not reach statistical significance (Fig. 4A). Analysis of beat-to-beat variability revealed a tendency toward increased irregularity in the SN-Sec treated group, consistent with emerging rhythm instability (Supplementary Fig. 6A).

To specifically evaluate calcium handling efficiency, normalized calcium transient kinetics were analyzed. Both the t-BHP and SN-Sec treated groups showed significantly prolonged time to 50% decay compared with control cells, with the most pronounced delay observed in the SN-Sec treated group (Fig. 4B). Similarly, calcium transient duration at 50% recovery was significantly extended in the SN-Sec treated group relative to the t-BHP group (Fig. 4B). In contrast, the rise time from 50% to peak calcium remained comparable across groups, indicating that calcium release kinetics were largely preserved (Supplementary Fig. 6B). Further analysis of late-phase calcium clearance demonstrated a marked prolongation of the 90% decay time specifically in the SN-Sec treated group (Supplementary Fig. 6C). This selective impairment of calcium reuptake resulted in a substantial increase in cytosolic calcium burden. Consistent with this, calcium transient amplitude increased stepwise across conditions, with the highest amplitude observed in the SN-Sec treated group (Fig. 4C). This elevation was accompanied by a significant expansion of the area under the calcium transient curve at 90% decay, indicating sustained intracellular calcium accumulation per beat (Fig. 4C).

We next examined electrophysiological properties to determine whether altered calcium dynamics were accompanied by changes in action potential behavior. Voltage recordings revealed a modest reduction in spontaneous firing rate under Two-Hit conditions, although this did not reach statistical significance (Fig. 4D). In contrast, action potential morphology in the SN-Sec treated group was distinctly altered. Normalized traces demonstrated a significant prolongation of action potential duration (APD90) in the SN-Sec treated group compared with the t-BHP group (Fig. 4E). Analysis of raw waveforms further revealed an increase in action potential amplitude in the SN-Sec treated group (Supplementary Fig. 6D).

Together, these findings demonstrate that sterile inflammatory stress following oxidative priming selectively impairs calcium reuptake kinetics and alters repolarization dynamics in hiPSC-CMs. The combination of delayed calcium clearance and prolonged action potential duration indicates the formation of an unstable electromechanical state under Two-Hit conditions.

### 3.5 Two-hit stress disrupts calcium regulatory balance and promotes maladaptive structural remodeling in human heart organoids

**Fig. 5:**
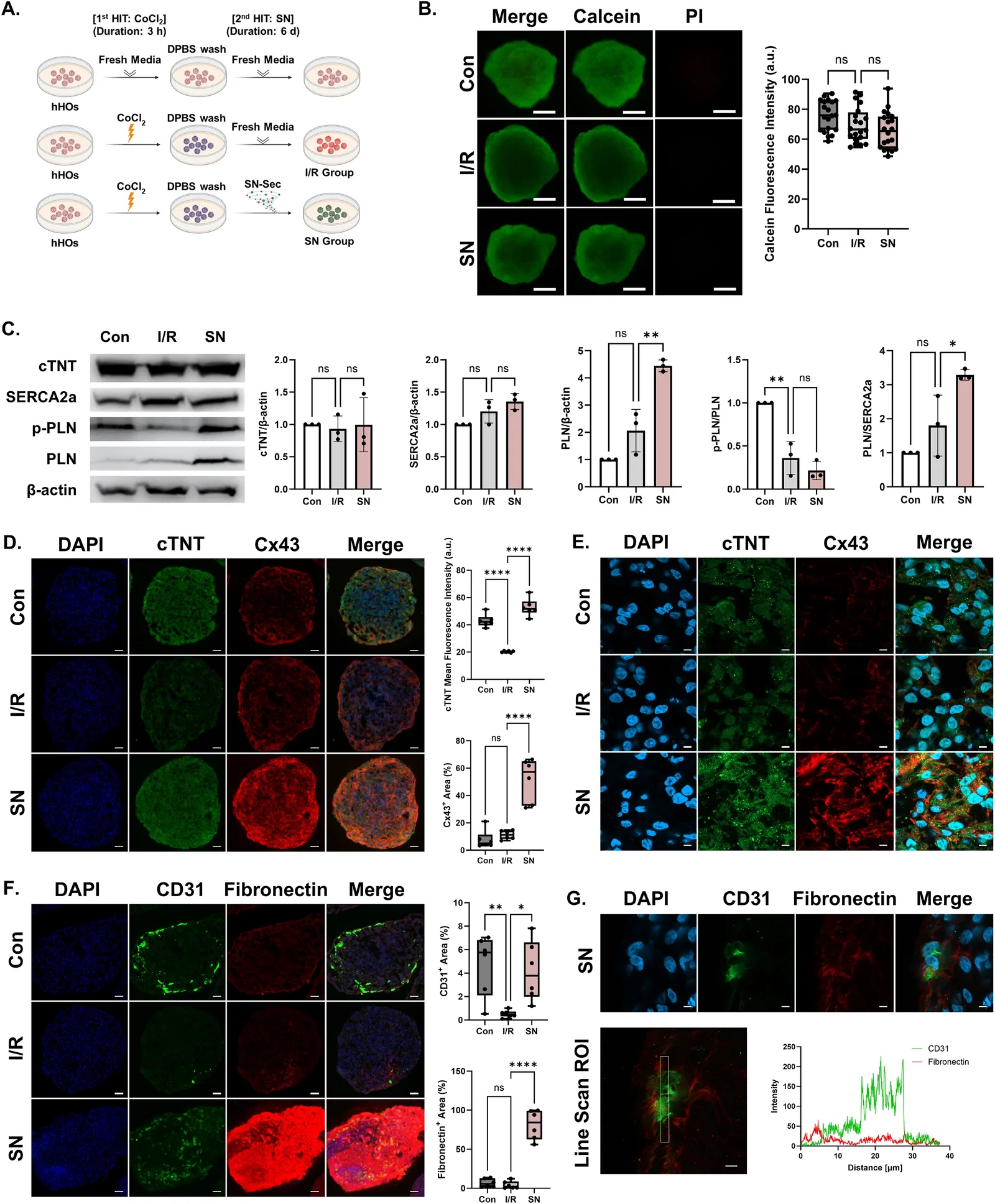
**Analysis of calcium regulatory protein expression and tissue-level extracellular matrix remodeling markers in human heart organoids under Two-Hit stress conditions.** **A.** Schematic illustration of experimental design. Human heart organoids (hHOs) were treated with vehicle (Control), CoCl_2_ followed by fresh media (I/R), or CoCl_2_ followed by SN-Sec (SN). **B.** Representative live/dead staining images using Calcein-AM (Green) and PI (Red). The bar graph quantifies the viable tissue volume. **C.** Western blot analysis of structural protein (cTNT) and calcium handling proteins (SERCA2a, Total PLN, p-PLN). Bar graphs quantify the expression levels of cTNT, SERCA2a, and Total PLN (normalized to β-actin), followed by the phosphorylation ratio (p-PLN/Total PLN) and the stoichiometric ratio (Total PLN/SERCA2a), highlighting the imbalance in calcium-regulatory proteins correlated with diastolic dysfunction. **D.** Representative immunofluorescence images of cardiomyocytes (cTNT) and gap junctions (Cx43). Bar graphs show the quantification of cTNT fluorescence intensity and % Area of Cx43 per section. **E.** Representative high-magnification confocal images visualizing nuclei (DAPI), cardiomyocytes (cTNT), and gap junctions (Cx43), alongside the merged image. **F.** Representative immunofluorescence images of endothelial cells (CD31) and extracellular matrix deposition (Fibronectin). Bar graphs show the % Area of CD31 and % Area of fibronectin per section as an indicator of adverse tissue remodeling. **G.** Representative high-magnification confocal images of the SN-Sec treated group showing DAPI, CD31, and Fibronectin, along with the merged view. The panel includes a line scan analysis of the indicated Region of Interest (ROI) and the corresponding fluorescence intensity profile demonstrating co-localization. Data are presented as Mean ± SEM from 3 independent experiments. Images are representative of these experiments. ns: not significant, *P < 0.05, **P < 0.01, ****P < 0.0001 vs. I/R group. Scale bars = 100 μm (B, D, F) and 5 μm (E, G).

To validate the Two-Hit phenotype in a multicellular tissue context, we next examined human iPSC-derived heart organoids (hHOs), which integrate cardiomyocytes, endothelial cells, and stromal populations. CoCl_2_ efficiently induced myocardial infarction and fibrosis in hHOs [25]. To model the ischemic component of ischemia/reperfusion injury in this three-dimensional system, hHOs were exposed to the hypoxia mimetic CoCl_2_ as the initial insult, followed by treatment with SN-Sec to induce sterile inflammation (Fig. 5A).

**Fig. 6:**
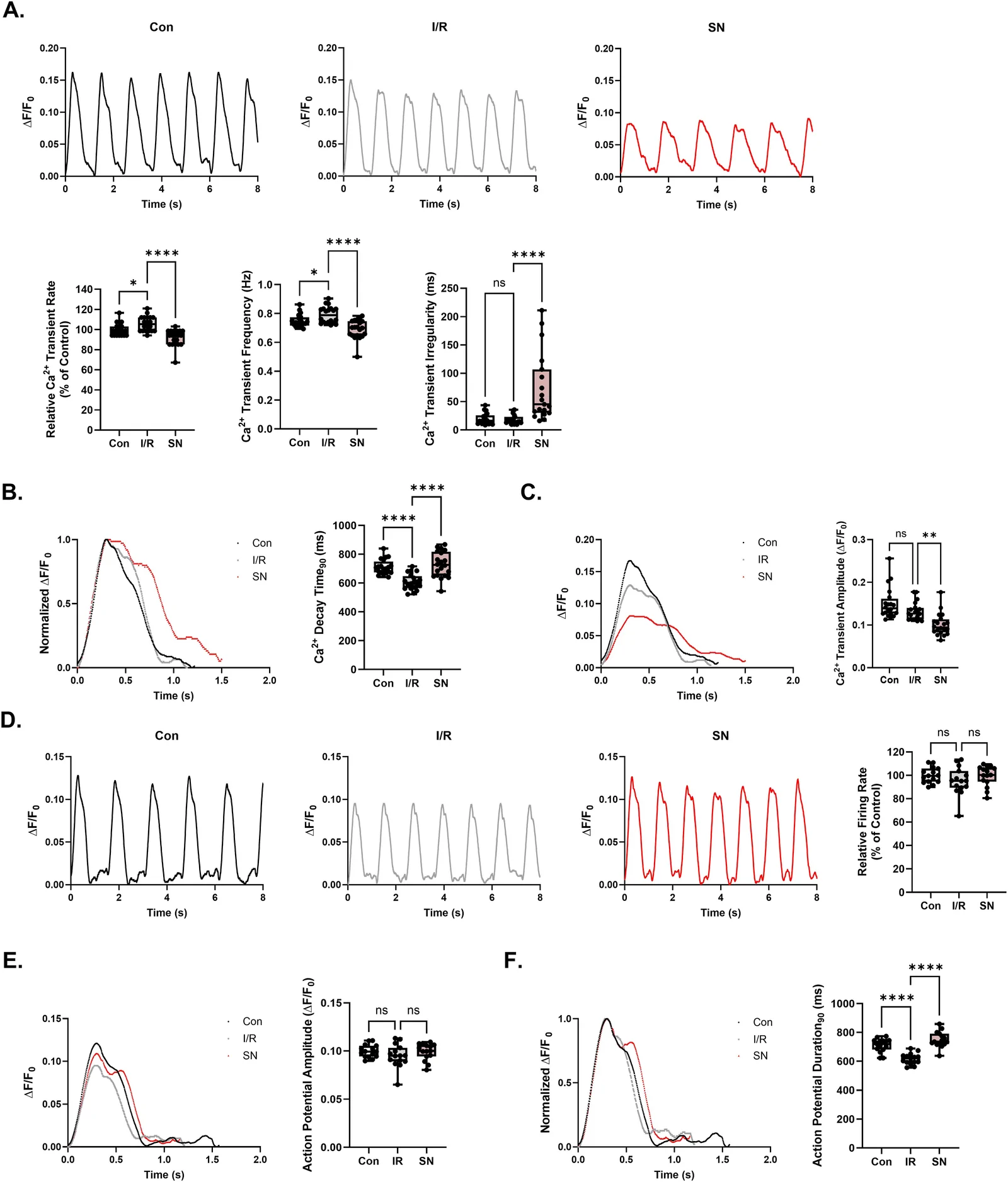
**Analysis of calcium handling dynamics and repolarization properties in human heart organoids under Two-Hit stress conditions.** **A.** Representative spontaneous calcium transient traces (ΔF/F_0_) recorded in human heart organoids (hHOs) using Fluo-4 AM over an 8 s duration. Bar graphs display the Ca^2+^ transient rate (BPM), Ca^2+^ transient frequency (Hz), and Ca^2+^ transient irregularity (ms). **B.** Representative normalized single transient trace. The bar graph quantifies the Ca^2+^ decay time_90_. **C.** Representative raw single transient trace (ΔF/F_0_). The bar graph displays the Ca^2+^ transient amplitude (ΔF/F_0_). **D.** Representative optical action potential traces (ΔF/F_0_) recorded in hHOs using Di-4-ANEPPS over an 8 s duration. The bar graph displays the firing rate (BPM). **E.** Representative raw single action potential trace (ΔF/F_0_). The bar graph quantifies the action potential amplitude (ΔF/F_0_). **F.** Representative normalized single action potential trace. The bar graph quantifies the action potential duration at 90% repolarization (APD_90_). Data are presented as Mean ± SEM from 5 independent experiments. Traces are representative of these experiments. ns: not significant, *P < 0.05, **P < 0.01, ***P < 0.001, vs. t-BHP group.

Upregulation of hypoxia-inducible factor 1α (HIF-1α) confirmed effective induction of hypoxic stress in hHOs by CoCl_2_ treatment (Supplementary Fig. 7A). Upon withdrawal of CoCl_2_, hHOs exhibited a pronounced increase in intracellular (DCFDA) and mitochondrial (MitoSOX) reactive oxygen species, indicating redox imbalance during the reperfusion phase (Supplementary Fig. 7B). Live/dead staining revealed that overall organoid viability was preserved across experimental groups. Quantitative analysis of calcein fluorescence intensity showed a modest decreasing trend following injury, although these changes did not reach statistical significance (Fig. 5B). These findings indicate that the applied stress conditions might induce sublethal injury while maintaining gross tissue architecture.

We next examined the molecular basis of calcium handling dysfunction in hHOs at the tissue level. Protein expression of SERCA2a and cardiac troponin T (cTNT) remained unchanged across the control, I/R, and SN-Sec treated groups (Fig. 5C). In contrast, marked alterations were observed in PLN regulation. While the ratio of phosphorylated PLN to total PLN was similarly reduced in both the I/R and SN-Sec treated groups, total PLN protein levels were selectively and significantly increased in the SN-Sec treated group (Fig. 5C). As a result, the PLN/SERCA2a ratio was markedly elevated under sterile inflammatory conditions, indicating a stoichiometric shift that associated with delayed calcium reuptake.

We subsequently assessed structural remodeling within the organoids. Wide-field immunofluorescence imaging revealed an apparent reduction in cTNT signal intensity in the I/R group, whereas Cx43 expression remained comparable to controls (Fig. 5D). However, hHOs in the SN-Sec treated group exhibited pronounced structural compactions, characterized by increased cTNT signal density and abnormal accumulation of Connexin 43 (Cx43) (Fig. 5D). Notably, high-resolution confocal analysis (Fig. 5E) revealed that these accumulated Cx43 signals were spatially disorganized and coarse, contrasting with the discrete punctate gap junction plaques observed in the control group. Additionally, three-dimensional Z-stack reconstruction further confirmed widespread intracellular retention of Cx43 throughout the organoid volume (Supplementary Video 1).

Supplementary data related to this article can be found online at https://doi.org/10.1016/j.mtbio.2026.103634

The following are the Supplementary data related to this article:

Multimedia component 2

We further examined the vascular compartment and extracellular matrix remodeling. The I/R injury led to a marked loss of CD31-positive endothelial structures compared with the control group. In contrast, the SN-Sec treated group preserved the endothelial network (Fig. 5F). Notably, this vascular preservation was accompanied by a pronounced accumulation of fibronectin, which was absent in the control group. High-resolution confocal imaging and three-dimensional reconstruction demonstrated spatial co-localization of fibronectin deposition with CD31-positive structures (Fig. 5G; Supplementary Video 2), indicating that endothelial niches are closely associated with fibrotic matrix accumulation under Two-Hit stress.

Supplementary data related to this article can be found online at https://doi.org/10.1016/j.mtbio.2026.103634

The following are the Supplementary data related to this article:

Multimedia component 3

Together, these data demonstrate that Two-Hit stress in a tissue-level cardiac model induces a maladaptive remodeling state distinct from the predominant structural loss in the I/R group, characterized by disruption of calcium regulatory balance through PLN accumulation, aberrant gap junction organization, and vascular-associated fibrotic remodeling.

### 3.6 Two-hit stress induces diastolic calcium failure and electromechanical discordance in human heart organoids

Excessive interstitial fibrosis and aberrant Cx43 distribution are closely associated with cardiac conduction disturbances [46,47]. To determine whether the molecular and structural remodeling observed in hHOs translates into functional impairment at the tissue level, we assessed calcium handling dynamics and electrophysiological properties using high-speed optical mapping. Intracellular calcium transients were recorded using Fluo-4 AM, and membrane potential dynamics were evaluated with the voltage-sensitive dye Di-4-ANEPPS.

Analysis of calcium transients revealed a biphasic pattern of rhythmic contractile activity under Two-Hit stress. While the I/R injury group showed a modest increase in spontaneous beating frequency relative to the control group, subsequent exposure to SN-Sec significantly suppressed calcium transient frequency (Fig. 6A). This reduction was accompanied by a pronounced increase in beat-to-beat irregularity, indicating impaired pacing stability under Two-Hit conditions (Fig. 6A). Detailed kinetic analysis demonstrated a selective impairment in calcium reuptake. The time to 90% decay of calcium transients was significantly prolonged exclusively in the SN-Sec treated group compared with both the control and the I/R groups (Fig. 6B). In contrast, calcium transient amplitude was significantly reduced in the SN-Sec treated group, whereas the I/R group did not show altered amplitude (Fig. 6C). These findings indicate impaired diastolic calcium clearance and reduced systolic calcium availability in hHOs exposed to Two-Hit stress.

We next examined electrophysiological properties to evaluate excitation dynamics. Voltage-sensitive dye recordings revealed that spontaneous electrical firing rates remained elevated in the SN-Sec treated group, comparable to those observed in the I/R group (Fig. 6D). In contrast to the suppressed calcium cycling frequency, this preserved electrical activity suggests a divergence between excitation and calcium-dependent contraction under Two-Hit stress. Assessment of action potential morphology further revealed altered repolarization dynamics. The I/R group exhibited a reduction in action potential amplitude and shortened action potential duration (APD90) compared to the control group (Fig. 6E and F). In contrast, the SN-Sec treated group maintained action potential amplitude and APD90 values comparable to the control group, resulting in a relative prolongation of repolarization compared with the I/R group (Fig. 6F).

Collectively, these data demonstrate that sterile inflammation following ischemic injury induces a profound electromechanical discordance, where suppressed calcium cycling and delayed decay coexist with preserved electrical firing and prolonged action potential duration.

## 4 Discussion

In this study, we established a human-relevant Two-Hit model that integrates oxidative stress (Hit 1: t-BHP or CoCl_2_) with the secretome of M1 macrophages challenged by secondary necrosis (Hit 2: SN-Sec) to recapitulate key features of myocardial ischemia/reperfusion (MI/R) injury. Unlike conventional models relying solely on chemical ROS inducers, this system mimics the critical phase of efferocytotic overload, in which uncleared apoptotic cells sustain a pro-oxidant sterile inflammatory response [13,48,49]. Our findings demonstrate that this inflammatory microenvironment does not simply accompany tissue injury but actively contributes to pathological remodeling characterized by fibrosis, diastolic dysfunction, and arrhythmogenic vulnerability.

In establishing Hit 1 across our platforms, distinct primary stress agents were selected to accommodate the structural complexity of 2D versus 3D systems. In 2D monolayers, acute pulse treatment with t-BHP enabled precise and direct titration of reactive oxygen species to interrogate single-cell redox vulnerability and rapid signaling cascades. In contrast, for 3D heart organoids, CoCl_2_ was utilized as a hypoxia mimetic to induce robust HIF-1α stabilization and replicate tissue-level core ischemia throughout the dense multicellular architecture, as described in our previous study [25], thereby overcoming diffusion gradients associated with short-term chemical oxidants. While t-BHP and CoCl_2_ engage distinct upstream primary pathways (direct chemical oxidation versus hypoxia signaling), both converged on a robust ROS surge during the subsequent reperfusion phase, providing a physiologically consistent oxidative and ischemic priming prior to exposure to the inflammatory secretome (Hit 2).

A major finding of this study is the cell-type-specific divergence in response to sequential Two-Hit stress. Endothelial cells exhibited pronounced susceptibility, coinciding with exhaustion of the inducible antioxidant enzyme HO-1 and subsequent cell death [50]. These observations underscore the vulnerability of the vascular compartment to combined oxidative and inflammatory insults. In contrast, hiPSC-derived cardiomyocytes preserved viability under the same conditions, reflecting their superior intrinsic antioxidant capacity. However, this survival was accompanied by maladaptive remodeling [51,52]. Specifically, SN-Sec induced upregulation of α-SMA in cardiomyocytes, indicating stress-induced cytoskeletal remodeling or a regression toward a partial dedifferentiated state. Together, these findings suggest that the interplay between oxidative priming and sterile inflammation promotes cardiomyocyte survival at the expense of functional integrity, thereby contributing to pathological remodeling of the myocardium [53].

To capture the temporal evolution of inflammation-driven remodeling, we employed both 2D monolayer cultures and 3D heart organoids. These platforms represent complementary stages of disease progression, with the 2D system reflecting early cellular responses and the 3D organoid model recapitulating tissue-level consolidation. In the 2D hiPSC-CM model, short-term inflammatory exposure was associated with rapid loss of Cx43, consistent with early gap junction disassembly triggered by inflammatory stress [54]. By contrast, the 3D organoid system revealed more complex remodeling outcomes, enabled by multicellular interactions that cannot be modeled in 2D culture systems of single cell type.

Within the vascularized microenvironment of human heart organoids, Cx43 exhibited a striking pattern of redistribution rather than uniform loss. While acute ischemic injury is commonly associated with Cx43 degradation, chronically failing myocardium often displays aberrant accumulation of non-junctional Cx43, resulting in apparent signal expansion despite impaired electrical coupling. Such redistribution has been attributed to redox-dependent trafficking defects [55]. In line with this concept, the diffuse cytosolic accumulation of Cx43 observed in SN-Sec treated organoids likely reflects disrupted trafficking and structural disorganization associated with prolonged inflammatory stress [56].

In parallel with gap junction remodeling, SN-Sec-exposed organoids exhibited a distinctive pattern of vascular preservation coupled with fibrotic progression. Whereas I/R injury alone resulted in marked loss of CD31-positive endothelial structures, the Two-Hit stress preserved the endothelial network while promoting fibronectin deposition. Notably, fibrotic matrix accumulation was spatially associated with preserved vascular structures, suggesting that endothelial niches may actively participate in fibrotic remodeling under inflammatory conditions. This vascular–interstitial crosstalk may underlie the “survival with scarring” phenotype observed in the post-ischemic myocardium [57,58].

At the mechanistic level, our data identify dysregulation of calcium handling through the PLN axis as a critical molecular feature associated with inflammation-induced diastolic dysfunction. In contrast to chronic heart failure models characterized by transcriptional downregulation of SERCA2a [59], Two-Hit stress in our system selectively induced accumulation of total PLN while preserving SERCA2a expression. This stoichiometric imbalance increased the PLN/SERCA2a ratio, which is associated with impaired sarcoplasmic reticulum calcium reuptake [60]. Functionally, this manifested as prolonged calcium decay kinetics, impaired diastolic relaxation, and reduced contractile efficiency, highlighting a distinct pathological window in which calcium handling is disrupted primarily by inhibitor accumulation rather than pump loss.

High-speed optical mapping further revealed pronounced electromechanical discordance in organoids under Two-Hit conditions. Although electrical automaticity remained elevated, intracellular calcium cycling was markedly slowed, reflecting the inhibitory effect of PLN accumulation on calcium reuptake. In addition, SN-Sec-treated organoids failed to exhibit the adaptive shortening of action potential duration typically observed following I/R injury, resulting in relative repolarization prolongation [61]. The combination of delayed calcium clearance, impaired repolarization, and disrupted cellular connectivity creates a highly arrhythmogenic substrate within the myocardium [62].

Furthermore, the phenotypic features captured by our Two-Hit system, such as preserved cell and tissue viability amidst severe diastolic calcium handling failure, interstitial fibrosis, and vascular-associated matrix deposition, align with the clinical hallmarks of heart failure with preserved ejection fraction (HFpEF) [63]. HFpEF is increasingly recognized as a systemic syndrome associated with chronic inflammation and microvascular dysfunction; however, physiological human in vitro models that recapitulate this survival with diastolic stiffening or dysfunction axis have remained scarce. By incorporating a secondary necrosis-driven inflammatory secretome, our human Two-Hit cardiac platform could provide a translationally relevant model not only for post-ischemic remodeling, but also for dissecting inflammation-driven HFpEF pathophysiology and evaluating targeted therapeutic strategies.

While our Two-Hit human platform provides valuable translational insights into post-ischemic remodeling, certain limitations should be noted. First, the use of THP-1 and Jurkat cells serves as a standardized biomimetic proxy for failed efferocytosis rather than replicating the exact multi-lineage primary DAMP profile of the infarcted human heart. Second, HUVECs represent a macrovascular endothelial cell type, substituting them with human coronary artery or cardiac microvascular endothelial cells in future organoid models will better simulate tissue-specific vascular dynamics. Lastly, although a strong stoichiometric correlation was identified between PLN accumulation and delayed calcium decay, direct genetic perturbation studies will be required to definitively dissect the individual causal contribution of the PLN axis to this diastolic dysfunction.

Nonetheless, the human-derived, SN-Sec–based platform described here provides a versatile approach for modeling sterile inflammation across diverse pathological contexts. Unlike co-culture systems relying on early apoptotic cells, which often elicit anti-inflammatory silent clearance [64], this model successfully captures the pathological consequences of failed efferocytosis, including membrane rupture and DAMP release. Therefore, it may be broadly applicable to the study of inflammatory remodeling in myocardial infarction, ischemic stroke, acute kidney injury, and autoimmune disorders. Moreover, the Two-Hit framework highlights a therapeutic window in which targeting efferocytosis, inflammatory signaling, or calcium regulatory pathways may prevent the transition from acute injury to chronic organ dysfunction.

## 5 Conclusion

In summary, this study establishes a physiologically relevant in vitro platform that captures key features of post-ischemic sterile inflammation. By integrating human iPSC-derived cardiac models with a secondary necrosis-derived inflammatory secretome, we recapitulated a survival with scarring phenotype that is often overlooked in conventional ischemia/reperfusion models.

We demonstrate that redox-driven efferocytotic failure elicits a pro-inflammatory secretome that is associated with disrupted calcium homeostasis, most notably inducing diastolic dysfunction correlated with dysregulation of the phospholamban/SERCA2a axis. This inflammatory milieu is further accompanied by electromechanical discordance in human heart organoids, revealing a high arrhythmogenic potential associated with sterile inflammation. Importantly, unlike standard ischemia/reperfusion models characterized by vascular loss, the Two-Hit organoid system exhibited preserved endothelial networks that coincided with pronounced interstitial fibrosis, highlighting a paradoxical but clinically relevant remodeling pattern.

Collectively, these findings position the Two-Hit human heart organoid model as a robust and translational platform for dissecting inflammation-driven cardiac dysfunction and for evaluating therapeutic strategies aimed at preventing heart failure and arrhythmia following myocardial infarction.

## CRediT authorship contribution statement

**So-Eun Jeong:** Writing – original draft, Investigation, Formal analysis, Data curation, Conceptualization. **Choongseong Han:** Methodology. **Jong-Hoon Kim:** Writing – review & editing, Conceptualization. **Dong-Hun Woo:** Writing – review & editing, Supervision, Resources, Funding acquisition.

## Declaration of generative AI and AI-assisted technologies in the writing process

During the preparation of this work, the authors used Gemini (Google) to improve language and readability and to assist in drafting the manuscript. After using this tool, the authors reviewed and edited the content as needed and took full responsibility for the content of the publication.

## Declaration of competing interest

The authors declare that they have no known competing financial interests or personal relationships that could have appeared to influence the work reported in this paper.

## Data Availability

Data will be made available on request.
