# Supplementary material for: A human two-hit platform modeling post-ischemic sterile inflammation and diastolic dysfunction in hiPSC-derived cardiac models
Source: Mater Today Bio. 2026 Sep 4;40:103634. doi: 10.1016/j.mtbio.2026.103634 (PMC13578629; doi:10.1016/j.mtbio.2026.103634)
Supplement: Supplementary file 1 [file mmc1.pdf]

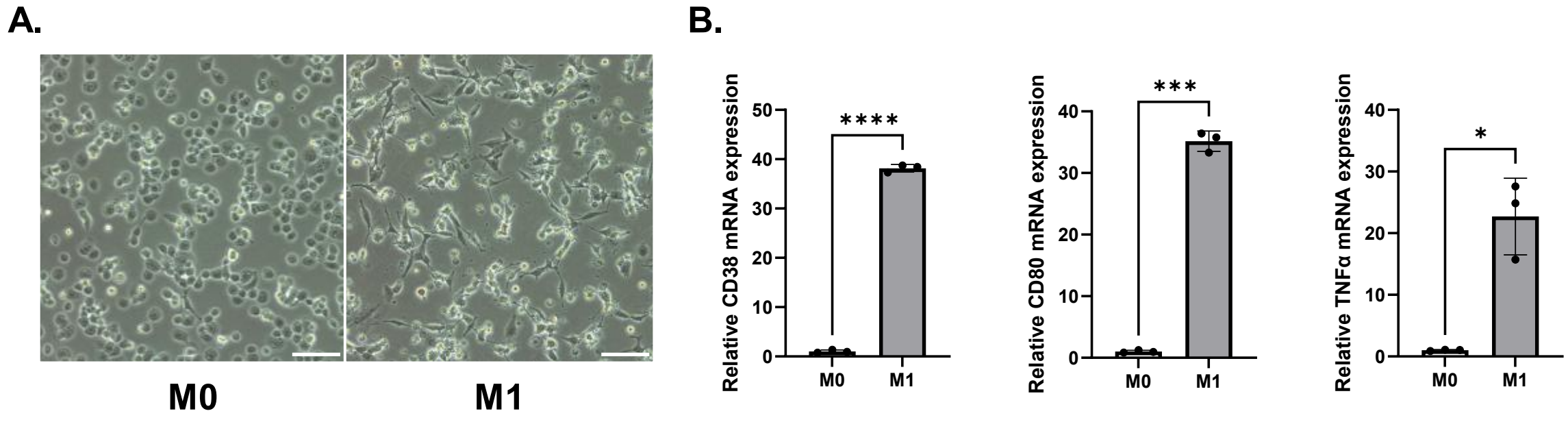

Supplementary Figure 1. Verification of M1 macrophage polarization

A. Representative brightfield images of M0 (unpolarized) and M1 (polarized) macrophages. **B.** Relative mRNA expression levels of the M1-specific markers CD38, CD80, and TNFα determined by RT-qPCR. Scale bar = 100 μm. Data are presented as mean ± SEM (n=3). $^*P < 0.05$, $^{***}P < 0.001$, $^{****}P < 0.0001$ vs. M0.

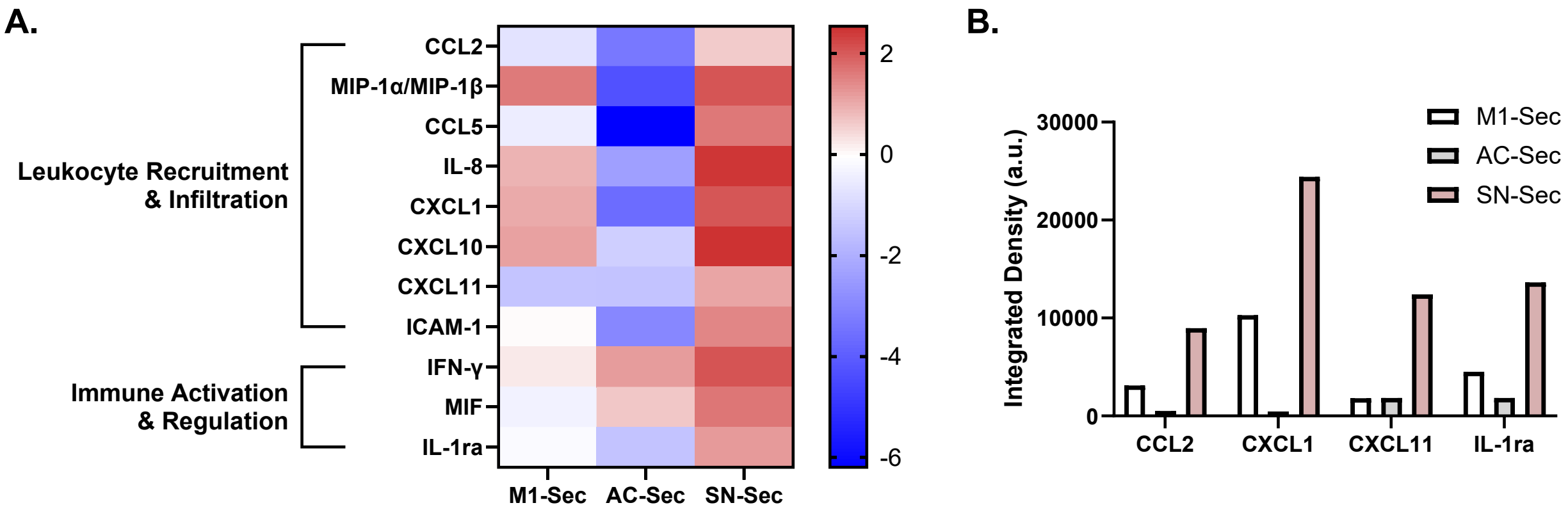

Supplementary Figure 2. Comprehensive analysis of secretome profiles

A. Heatmap representation of the cytokine array profiles across M1-Sec, AC-Sec, and SN-Sec groups. **B.** Cytokine array profiling. The bar graph represents the densitometric quantification of the array spots.

A.

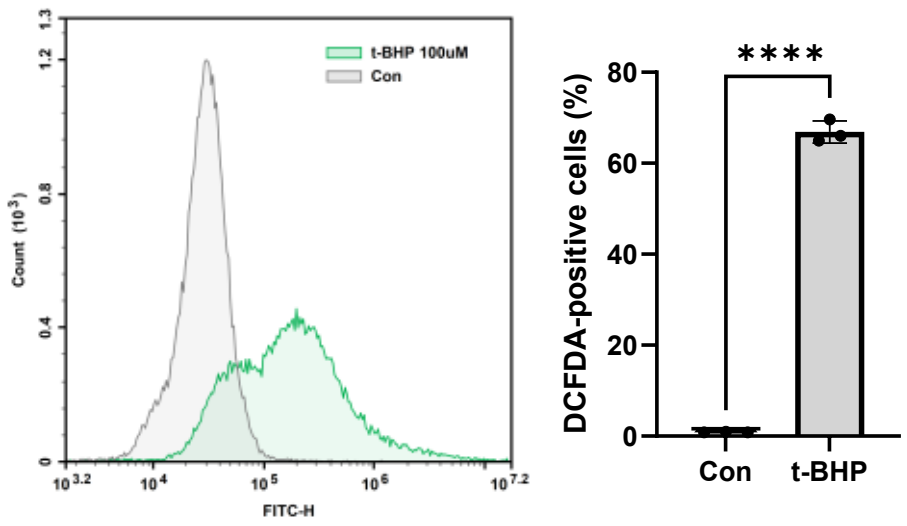

Supplementary Figure 3. Verification of t-BHP-induced oxidative stress in HUVECs

A. Flow cytometric assessment of intracellular ROS levels using DCFDA staining. HUVECs were treated with 100 μM t-BHP for 20 min. The left panel shows a representative histogram of DCFDA fluorescence. The right panel displays the quantitative analysis of the Mean Fluorescence Intensity (MFI). Data are presented as Mean ± SEM (n=3). ****$P < 0.0001$ vs. Control.

A.

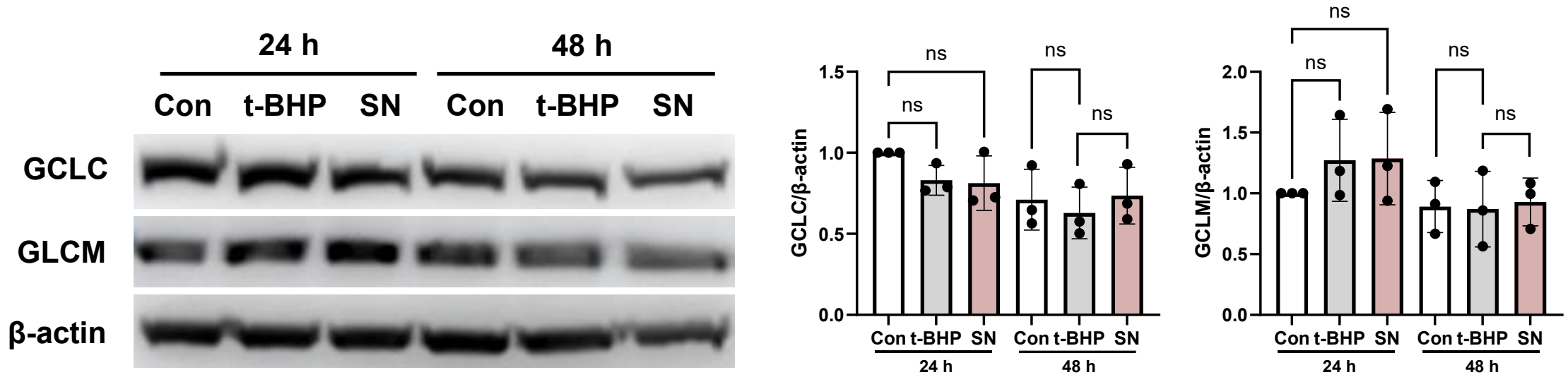

Supplementary Figure 4. Expression of the antioxidant enzymes GCLC and GCLM in HUVECs

A. Representative Western blot images showing the protein levels of GCLC and GCLM in HUVECs treated with t-BHP or SN-Sec (SN) for 24 and 48 h (normalized to β-actin). The bar graphs represent the densitometric quantification of GCLC and GCLM normalized to β-actin. Data are presented as Mean ± SEM (n=3). ns, not significant, vs. corresponding t-BHP.

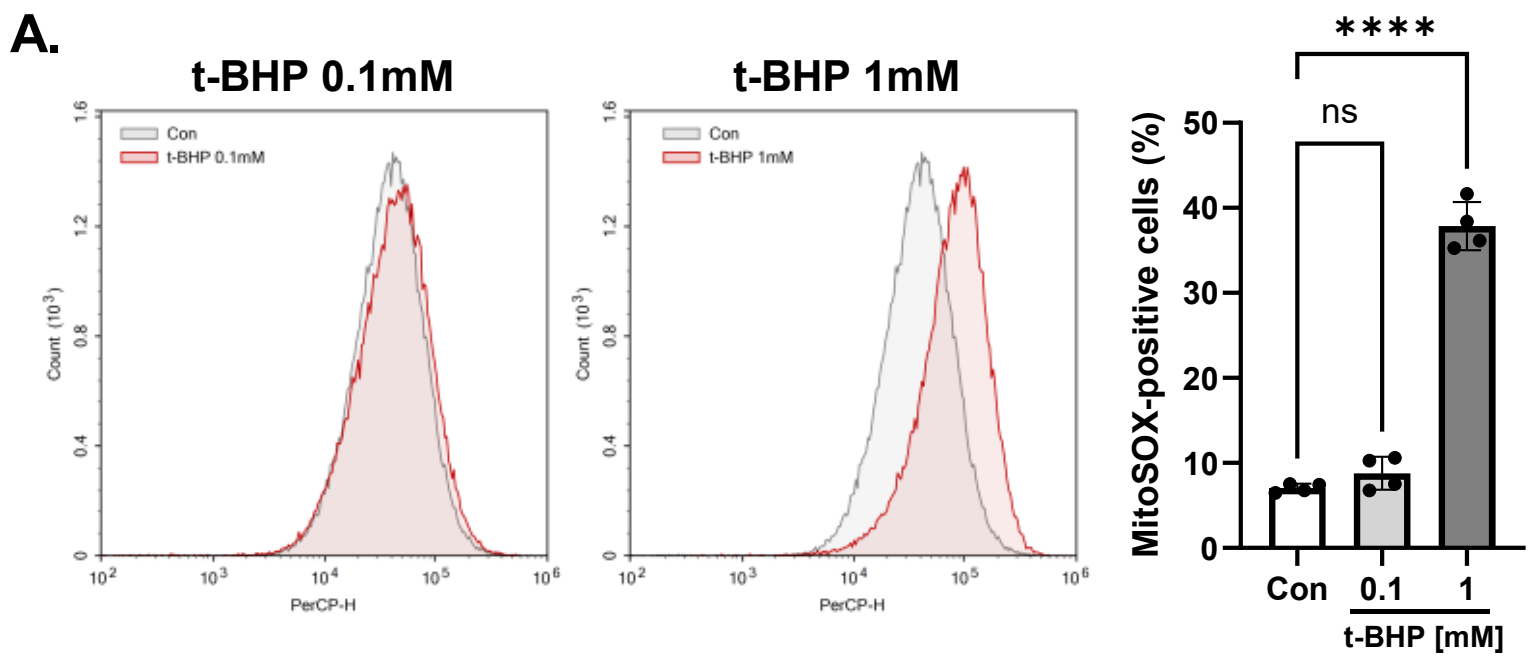

Supplementary Figure 5. Mitochondrial ROS generation in 2D human iPSC- derived cardiomyocyte monolayers

A. Flow cytometric assessment of mitochondrial ROS levels using MitoSOX Red staining. 2D iPSC-CMs were treated with t-BHP at concentrations of 0.1 mM and 1 mM for 20 min. The left panels display representative histograms of fluorescence intensity. The right panel shows the quantitative analysis of the Mean Fluorescence Intensity (MFI). Data are presented as Mean ± SEM (n=4). ns, not significant, ****$P < 0.0001$ vs. Control.

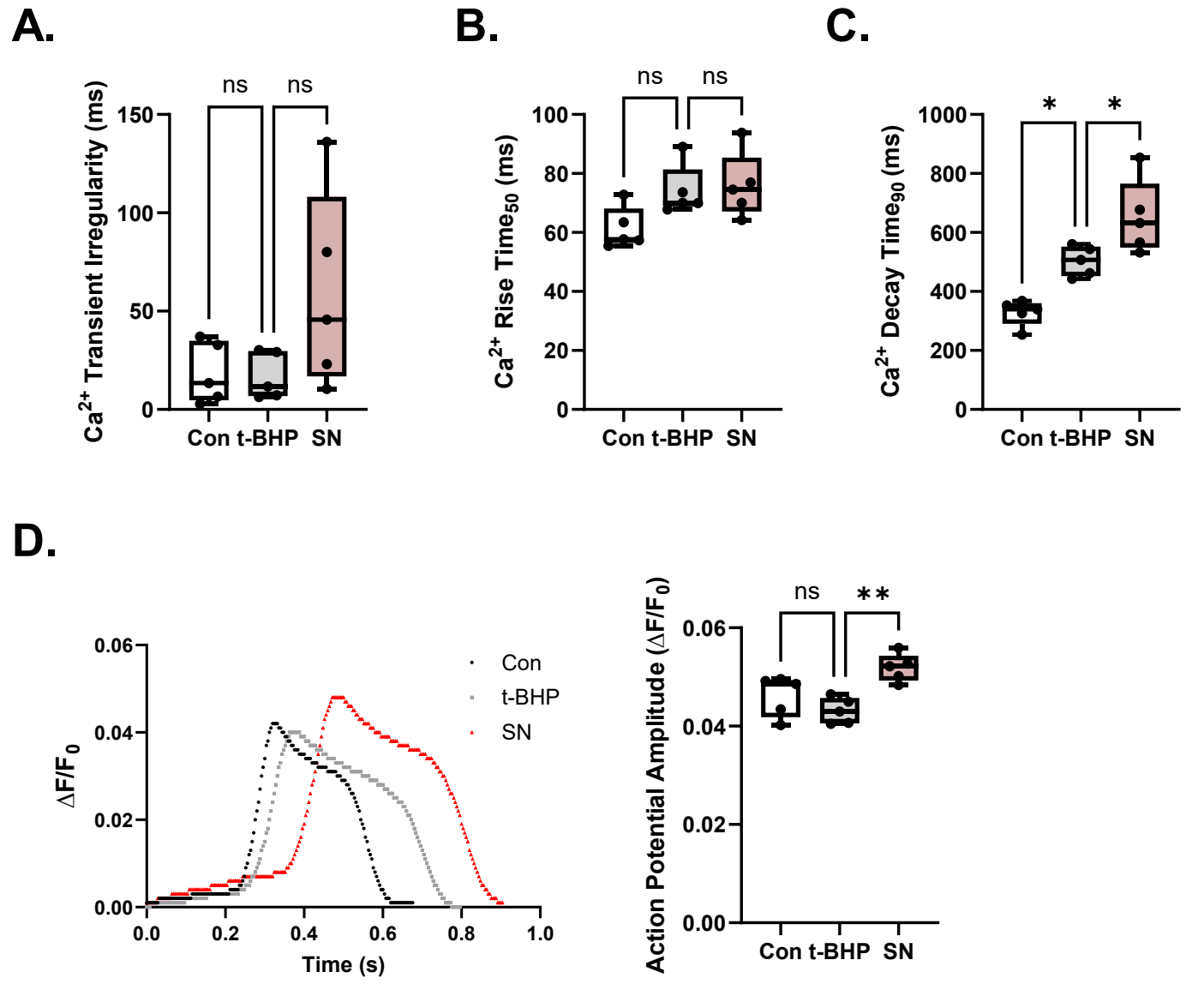

Supplementary Figure 6. Extended Kinetic Analysis of Calcium Transients and electrophysiological properties in hiPSC-CMs

A. Quantification of calcium transient irregularity. **B.** Quantification of calcium rise time to 50% peak. **C.** Quantification of calcium decay time to 90% baseline. **D.** Analysis of action potential. Representative optical voltage traces (left) and quantification of action potential amplitude (right). Data are presented as Mean ± SEM from 5 independent experiments. ns: not significant, $^*P < 0.05$, $^{**}P < 0.01$, vs. t-BHP.

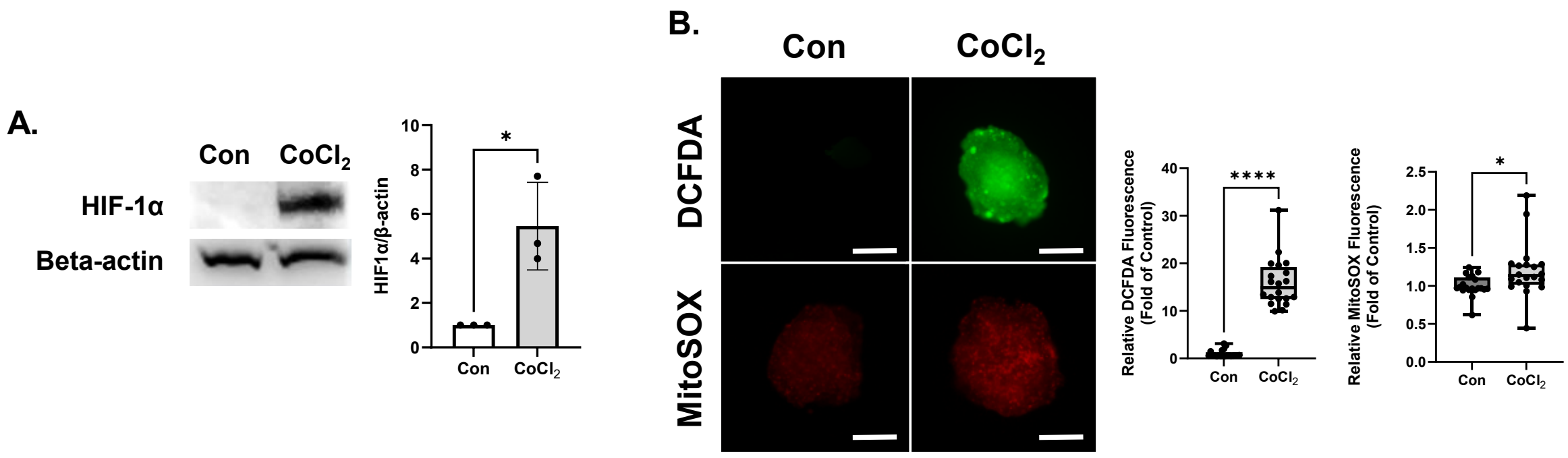

Supplementary Figure 7. Validation of hypoxia induction and oxidative stress in human heart organoids (hHOs)

A. Western blot analysis of Hypoxia-inducible factor 1-alpha (HIF-1α) in hHOs treated with $CoCl_2$ for 3 h (normalized to β-actin). **B.** Assessment of oxidative stress in hHOs treated with $CoCl_2$ for 3 h. Representative fluorescence images of whole organoids stained with DCFDA (green, intracellular ROS) and MitoSOX Red (red, mitochondrial superoxide). Scale bar = 100 μm. Data are presented as Mean ± SEM from 3 independent experiments. ns: not significant, **P* < 0.05, ****P < 0.0001 vs. Control.

Supplementary Video Legends

Supplementary Video 1. Three-dimensional reconstruction of Cx43 distribution in the SN group. The video displays a Z-stack 3D rendering of a human heart organoid exposed to SN-CM. Immunofluorescence staining visualizes cardiac troponin T (cTNT, green), connexin 43 (Cx43, red), and nuclei (DAPI, blue). The animation demonstrates the volumetric spatial distribution and intracellular accumulation of Cx43 signals within the organoid tissue.

Supplementary Video 2. Three-dimensional visualization of the fibro-vascular interface in the SN group. The video presents a Z-stack 3D reconstruction of the vascular network and extracellular matrix in the SN-Sec treated organoid. Immunofluorescence staining identifies endothelial cells (CD31, green), fibronectin (red), and nuclei (DAPI, blue). The rendering shows the spatial co-localization and structural arrangement of the endothelial network relative to the regions of fibronectin deposition.

Supplementary Table 1.

Target			
	M1-Sec	AC-Sec	SN-Sec
CCL1	1460.5	2319	5406
CCL2	3105	513	8969
MIP-1α/MIP-1β	15456	267	24559.5
CCL5	3673.5	69	18235
CD40 Ligand	647	359	2904
Complement Component C5	806	535	3229.5
CXCL1	10298	422.5	24412.5
CXCL10	11076.5	2247	33359.5
CXCL11	1818	1839.5	12409.5
CXCL12	981	1302	3674.5
G-CSF	1433.5	418	2840.5
GM-CSF	834	306.5	2876.5
ICAM-1	5119	664	16223
IFN-γ	5971	11804.5	24780
IL-1α	1381	580.5	3377.5
IL-1β	2100.5	1514	4884.5
IL-1ra	4505	1831	13644.5
IL-2	634	620	2256
IL-4	647.5	302.5	1718
IL-5	623	364	1947.5
IL-6	4239.5	627.5	4536.5
IL-8	9541.5	1003.5	32017
IL-10	631	389.5	2605.5
IL-12 p70	732.5	803.5	1662
IL-13	1313	2479.5	4959
IL-16	605.5	710	2234
IL-17A	343	335	1381.5
IL-17E	372	363.5	1858
IL-18	803	556	3381
IL-21	1033.5	424.5	4078
IL-27	427.5	254	1750.5
IL-32α	480	663	1396
MIF	3967	8143	18556.5
Serpin E1	1670.5	774	3726.5
TNF-α	840	347.5	2691.5
TREM-1	364	354	1234

Supplementary Method

Culture of Human Monocytic and T-cell Lines

The human monocytic cell line THP-1 (ATCC, TIB-202) and the human T-lymphocyte cell line Jurkat (Clone E6-1; ATCC, TIB-152) were maintained in RPMI-1640 medium (ATCC modification) supplemented with 10% fetal bovine serum (FBS) and 1% penicillin-streptomycin. For the optimal growth of THP-1 cells, 2-mercaptoethanol was added to a final concentration of 0.05 mM, as recommended by the manufacturer. Both cell lines were maintained in a humidified incubator at 37°C with 5% CO^2. The cells were cultured in suspension and maintained at a density between 1×10^5 cells/cm^2 and 1×10^6 cells/cm^2 viable cells/mL. Subculturing was performed every 2 to 3 days by adding fresh medium or by centrifugation followed by resuspension in new medium to ensure the cells did not exceed the maximum recommended density 1×10^6 cells/mL, which prevents the loss of differentiation potential and phenotypic shifts.

M1 Macrophage Differentiation and Activation

To induce macrophage differentiation, THP-1 cells were treated with 100 ng/mL phorbol 12-myristate 13-acetate (PMA; Sigma-Aldrich) for 48 hours in RPMI-1640 medium. This treatment promotes the transition from floating monocytes to adherent macrophage-like cells. Following the 48-hour induction, the PMA-containing medium was removed, and the cells were washed twice with Dulbecco's phosphate-buffered saline (DPBS) to eliminate non-adherent cells and residual PMA. For M1 polarization, the differentiated THP-1 macrophages were subsequently incubated for an additional 48 hours in fresh culture medium supplemented with 20 ng/mL recombinant human IFN-γ (R&D Systems) and 100 pg/mL lipopolysaccharide (LPS; Sigma-Aldrich). The successful transition to the M1 phenotype was confirmed by the observation of an elongated, amoeboid morphology and adherence to the culture vessel before being used for subsequent experiments or the collection of inflammatory secretomes.

RNA isolation and quantitative real-time PCR (RT-qPCR)

Total RNA was extracted from cells and organoids using TRIzol reagent (Invitrogen) according to the manufacturer’s instructions. The concentration and purity of RNA were determined using

a SpectraMax iD3 Multi-Mode Microplate Reader (Molecular Devices). Complementary DNA (cDNA) was synthesized using cDNA Synthesis Kit (Invitrogen). Quantitative real-time PCR (RT-qPCR) was performed using iQ™ SYBR® Green Supermix (Bio-Rad) on a CFX96 Touch Real-Time PCR Detection System (Bio-Rad). The relative mRNA expression levels were calculated using the $2^{-\Delta\Delta C_t}$ method and normalized to GAPDH. The primer sequences used for RT-qPCR are as follows:GAPDH (F: 5'- GTC TCC TCT GAC TTC AAC AGC G-3', R: 5'- ACC ACC CTG TTG CTG TAG CCA A-3'); CD38 (F:5'- TCT TGC CCA GAC TGG AGA AAG G-3', R: 5'- TGG ACC ACA TCA CAG GCA GCT T-3'); CD80 (F: 5'- CTC TTG GTG CTG GCT GGT CTT T-3', R: 5'- GCC AGT AGA TGC GAG TTT GTG C-3'); TNFα (F: 5'- CTC TTC TGC CTG CTG CAC TTT G-3', R: 5'- ATG GGC TAC AGG CTT GTC ACT C-3');

HUVEC Culture and Maintenance

Human Umbilical Vein Endothelial Cells (HUVECs; ATCC, CRL-1730) were maintained according to the manufacturer's instructions. The cells were cultured in Endothelial Cell Growth Medium. For the formulation, F-12K Medium (Kaighn's Modification, Gibco) was used as the basal medium, supplemented with 0.1 mg/mL of heparin and 0.05 mg/mL of endothelial cell growth supplement (ECGS), along with 10% fetal bovine serum (FBS).The cultures were maintained in a humidified incubator at 37°C with 5% CO2. For subculturing, cells were rinsed with TrypLE™ (Gibco) when they reached approximately 80% confluence to prevent overgrowth and loss of contact inhibition.

Annexin V-FITC/PI Staining for Apoptosis Analysis

To quantify the distribution of live, apoptotic, and necrotic cells, flow cytometry was performed using the Annexin V-FITC/PI Apoptosis Detection Kit (Thermo Fisher Scientific) according to the manufacturer's instructions. Following experimental treatments, cells (e.g., Jurkat cells or HUVECs or hiPSC-CMs) were harvested, washed with DPBS (Dulbecco's Phosphate Buffered Saline), and resuspended in 1X Annexin-Binding Buffer at a density of approximately 1×10^6 cells/mL. For each 100 μL of cell suspension, 5 μL of Annexin V-FITC and 1 μL of Propidium Iodide (PI) working solution (100 μg/mL) were added. The samples were incubated in the dark at room temperature for 15 minutes. After incubation, an additional 400 μL of 1X Annexin-Binding Buffer was added to each tube, and the samples were gently mixed and kept on ice. Fluorescence was measured using a flow cytometer (the ACEA NovoCyte-3000 Flow

cytometer, Agilent) within 1 hour to ensure maximal signal stability. The cell populations were categorized as follows: viable (Annexin V^{-}/PI^{-}), early apoptotic (Annexin V^{+}/PI^{-}), and late apoptotic/secondary necrotic (Annexin V^{+}/PI^{+}). Data were analyzed using NovoCyte software (Agilent).

Cytokine Profiling using Antibody Array

To simultaneously profile the secretion of multiple cytokines and chemokines in the culture supernatants, a Proteome Profiler Human Cytokine Array Kit (R&D Systems) was used according to the manufacturer's instructions. Briefly, the nitrocellulose membranes, which are pre-coated with 36 different cytokine capture antibodies in duplicate, were blocked with Array Buffer 4 for 1 hour on a rocking platform. During blocking, 500 μL of cell culture supernatant was mixed with Array Buffer 4 and Array Buffer 5 and then incubated with 15 μL of the Detection Antibody Cocktail at room temperature for 1 hour. The blocked membranes were then incubated with the sample/antibody mixture overnight at 4 ℃ Following three washes with 1X Wash Buffer, the membranes were incubated with Streptavidin-HRP for 30 minutes. The protein signals were visualized using Chemi Reagent Mix and captured using a chemiluminescence imaging system (ChemiDoc, Bio-Rad). The pixel intensity of each spot was quantified using ImageJ software. The average background signal from the negative control spots was subtracted from each cytokine spot, and the resulting values were normalized to the reference spots located at the corners of each membrane.

Western Blot Analysis

Total protein was extracted from [cells or organoids] using RIPA lysis buffer supplemented with protease and phosphatase inhibitors. Protein concentrations were determined using a Bradford protein assay kit (Thermo) according to the manufacturer's instructions. Equal amounts of protein were loaded and separated by SDS-PAGE and subsequently transferred onto nitrocellulose membranes. The membranes were blocked with 3% bovine serum albumin (BSA) in Tris-buffered saline with Tween-20 (TBST) for 1 hour at room temperature. The membranes were then incubated overnight at 4℃ with specific primary antibodies. After three washes with washing buffer, the membranes were incubated with appropriate horseradish peroxidase (HRP)-conjugated secondary antibodies for 1 hour at room temperature. The protein bands were visualized using an enhanced chemiluminescence (ECL) detection system

and captured with a digital imaging system. The intensity of the bands was quantified using ImageJ software and normalized to the expression of loading controls.

Cell Viability Assay

Cell viability was assessed using the Cell Counting Kit-8 (CCK-8; Dojindo Molecular Technologies) according to the manufacturer’s protocol. Briefly, cells were cultured in 96-well plates and subjected to the designated experimental treatments. Following treatment, CCK-8 solution (10 μl per 100 μl of medium) was added to each well, and the plates were incubated for 1 to 4 hours at 37 ℃ in a humidified 5% CO2 atmosphere. The absorbance was then measured at 450 nm using a SpectraMax iD3 Multi-Mode Microplate Reader (Molecular Devices). Cell viability was calculated as a percentage relative to the untreated control group after subtracting the background absorbance of the medium-only blanks.

Preparation and Histological Sectioning of Heart Organoids

For the histological analysis of 3D hHOs, the organoids were first fixed in 4% paraformaldehyde (PFA) at 4℃ overnight. To provide cryoprotection, the fixed organoids were dehydrated in a 30% sucrose in DPBS (Dulbecco's Phosphate Buffered Saline) until they sank to the bottom of the tube. The organoids were then embedded in Optimal Cutting Temperature (OCT) compound (Sakura Finetek) and rapidly frozen using liquid nitrogen or at -80℃. The embedded samples were sectioned at a thickness of 10 μm using a cryostat (Leica CM1860) and mounted onto glass slides.

Immunofluorescence Staining and fluorescence Imaging

For cell samples, cells were fixed with 4% paraformaldehyde (PFA) for 15 minutes at room temperature. To prevent non-specific binding and permeabilize the cells, samples were incubated in a blocking buffer consisting of 1% bovine serum albumin (BSA) and 0.5% Triton X-100 in PBS (PBST). The samples were then incubated with primary antibodies [Target-specific antibodies] diluted in blocking buffer overnight at 4 ℃. Following three washes with PBST, the samples were incubated with appropriate Alexa Fluor-conjugated secondary antibodies (Invitrogen), alongside 1 μg/mL DAPI for nuclear counterstaining. After a final series of washes, the samples were mounted using Fluoromount-G™ Mounting Medium (Invitrogen) to preserve fluorescence signals. Fluorescence imaging was performed using a Nikon Eclipse

Ti2 microscope (Nikon). For quantitative analysis, image preprocessing was conducted using Fiji (ImageJ) software.

Functional Optical Mapping of Calcium and Voltage Dynamics

To evaluate the electrophysiological properties, functional imaging was performed using the Nautilai system (CuriBio). The organoids were washed twice with Tyrode's buffer and subsequently stained with fluorescent indicators for calcium and membrane potential. For calcium imaging, hiPSC-CM or hHOs were incubated with 10 μM Fluo-4 AM (Invitrogen) in Tyrode's buffer for 30 minutes at 37 ℃. For voltage imaging, the organoids were stained with 5 μM Di-4-ANEPPS (Invitrogen) in Tyrode's buffer for 15 minutes at 37 ℃. After staining, the organoids were washed three times with fresh warm Tyrode's buffer to remove residual dye before imaging. High-speed fluorescence images were captured and analyzed using the Nautilai software (CuriBio).

Live/Dead Cell Viability Assay and Imaging

To visualize and quantify the viability of the 3D heart organoids (hHOs), Calcein-AM/Propidium Iodide (PI) staining was performed. The organoids were washed twice with Tyrode's buffer and then incubated in a staining solution containing 1 μM Calcein-AM (Invitrogen) and 1 μL of Propidium Iodide (PI) working solution (100 μg/mL) (Invitrogen) in Tyrode's buffer for 30 minutes at 37 ℃ in the dark. Calcein-AM was used to identify live cells via green fluorescence, while PI was used to label dead cells with red fluorescence. Following the incubation, the staining solution was replaced with fresh Tyrode's buffer to remove background signals. Fluorescence imaging was conducted while the organoids remained submerged in Tyrode's buffer to maintain their physiological structural integrity and prevent dehydration during the imaging process. Images were captured using a Nikon Eclipse Ti2 microscope (Nikon). The viability was determined by analyzing the ratio of Calcein-positive (live) areas to PI-positive (dead) areas using Fiji (ImageJ) software.
